# Mulberry, Gut Microbiota and Gut Functionality: Effects Shaped by Raw Material and Processing Methods

**DOI:** 10.3390/biom16070965

**Published:** 2026-06-30

**Authors:** Marta Maria Miszczak, Karolina Kłosowska-Buryło, Joanna Magdalena Pieczyńska, Monika Bielecka, Anna Prescha

**Affiliations:** 1 Department of Dietetics and Bromatology, Wroclaw Medical University, Borowska 211, 50-556 Wrocław, Poland; m.miszczak@umw.edu.pl (M.M.M.); joanna.pieczynska@umw.edu.pl (J.M.P.); anna.prescha@umw.edu.pl (A.P.); 2 Student Research Group “Nutri-Sfera”, Faculty of Pharmacy, Wroclaw Medical University, Borowska 211, 50-556 Wrocław, Poland; 3 Department of Pharmaceutical Biology and Biotechnology, Wroclaw Medical University, Borowska 211A, 50-556 Wrocław, Poland

**Keywords:** mulberry, *Morus alba*, *Morus nigra*, phytochemicals, gut microbiota, intestinal functionality, polysaccharides, polyphenols, fermentation, extraction, processing methods

## Abstract

Mulberry species (*Morus* spp.) provide phytochemically distinct plant materials in which leaves are typically characterized by high levels of iminosugars (notably 1-deoxynojirimycin), flavonols/flavones, and polysaccharides, whereas fruits—especially *Morus nigra*—contain substantial amounts of anthocyanins alongside other phenolic compounds and polysaccharides. Importantly, the composition and biological properties of mulberry-derived products depend not only on species and plant part (leaf vs. fruit), but also on preparation and processing variables, including drying, maceration, fermentation, and extraction, or fractionation strategy (e.g., aqueous vs. hydroalcoholic extracts or enriched fractions). Such technological factors may substantially influence the chemical composition, bioavailability, and functionality of mulberry-derived preparations and thereby modify their interactions with gut microbiota and host metabolic processes. Available preclinical studies indicate that mulberry leaf- and fruit-derived preparations can affect gut microbial composition or activity in experimental models of metabolic dysfunction. Reported findings frequently include enrichment of microbial taxa commonly regarded as beneficial, such as *Bifidobacterium*, *Lactobacillus*, and *Akkermansia*, normalization of dysbiosis-associated microbial patterns, and increased production of short-chain fatty acids, particularly acetate, propionate, and butyrate. These microbial changes are sometimes observed alongside improvements in metabolic parameters such as glucose regulation, lipid profile, adiposity, or inflammatory markers. However, reported responses differ across plant parts, species, and preparation approaches, indicating that phytochemical composition and processing strategy are likely to influence biological outcomes. Interpretation of the current evidence is limited by the predominance of non-human studies and by incomplete or inconsistent reporting of extract composition, processing conditions, and standardization procedures. These factors reduce comparability between studies and complicate mechanistic interpretation of microbiome-related effects. Overall, existing preclinical data support the possibility that mulberry-derived preparations may influence metabolic health through microbiota-associated pathways shaped by both botanical origin and preparative technology. Well-designed human intervention studies using chemically characterized and standardized preparations, together with comprehensive gut microbiome analyses, are needed to determine the translational relevance of these observations and to identify which mulberry-derived preparations offer the greatest potential for supporting gut and metabolic health.

## 1. Introduction

Mulberry (*Morus* L.) belongs to the *Moraceae* family and comprises several species, among which white mulberry (*Morus alba* L.), black mulberry *(Morus nigra* L.), red mulberry (*Morus rubra* L.), and *Morus indica* L. are frequently described in the literature [1]. Mulberry species have distinct geographical origins. While *M. alba* originates from East Asia and was widely disseminated through cultivation, particularly in connection with sericulture, other species such as *M. nigra* are native to Western Asia and the Mediterranean region, and *M. rubra* is native to North America. *M. indica* is often considered a variety or synonym of *M. alba* and is associated with South and Southeast Asia [2,3]. Mulberry has a long history of use as both a food and medicinal resource. These historical applications are primarily associated with *M. alba* and can be traced back to ancient Chinese medicine, where various parts of the plant—including leaves, fruits, and root bark—were described in early pharmacopoeias such as the Shennong Bencao Jing (ca. 1st–2nd century CE) [4]. In Asia, raw materials derived from this plant have been commonly used in treating respiratory conditions, coughs, and fever (leaf and root bark) [5]. Its fruits have traditionally been consumed fresh, while also being attributed with functions such as improving vision, supporting liver health, and alleviating conditions described in traditional medicine, including symptoms associated with metabolic imbalance and premature ageing [6]. Beyond East Asia, mulberry was also incorporated into traditional medical practices in the Mediterranean and Middle Eastern regions, where it was used to manage symptoms such as fever and digestive disorders, often involving species such as *M. nigra* [7]. In more recent decades, these materials have also been investigated and applied in the context of modern disease categories, including diabetes, hypertension, headaches, and diuretic effects [8].

Mulberry typically grows as a deciduous tree or woody perennial, characterized by fissured bark and a cylindrical stem, and can reach heights of up to 10–15 m under favorable conditions, particularly in *M. alba* [3]. The plant is now widely cultivated across diverse climatic regions worldwide due to its high ecological adaptability [9]. It can be grown in various forms, such as bush, dwarf, or standard tree systems [10]. Recent comparative analyses of chloroplast genomes have refined the phylogenetic relationships within the genus *Morus*, revealing substantial genetic divergence between species traditionally grouped under similar taxonomic categories. These findings underscore the importance of precise botanical identification when evaluating phytochemical composition and biological effects [11].

The biological activity of mulberry species is attributed to a diverse range of compounds, including polyphenols, flavonoids, anthocyanins, alkaloids (notably iminosugars such as 1-deoxynojirimycin, DNJ), and polysaccharides [12]. These compounds have been associated with antioxidant, anti-inflammatory, hypolipidemic, and glucose-lowering effects [8]. Importantly, many of them—particularly high-molecular-weight polyphenols and complex polysaccharides—are only partially absorbed in the upper gastrointestinal tract and may reach the colon, where they can interact with gut microbiota [13].

**Figure 1:**
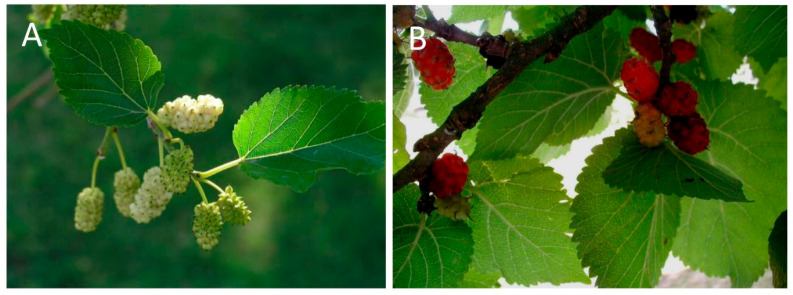
(**A**) *Morus alba* [https://commons.wikimedia.org/wiki/File:Morus-alba.jpg (accessed on 29 April 2026)]; (**B**) *Morus nigra* [https://commons.wikimedia.org/wiki/File:Morus-nigra.JPG (accessed on 29 April 2026)].

This growing body of evidence, together with the long-standing traditional use of mulberry, has contributed to increasing scientific and commercial interest in this plant in recent years. Both *M. alba* and *M. nigra* fruits have gained recognition as a nutraceutical and functional food ingredient (Figure 1). In particular, *M. alba* is now widely available in the form of leaf extracts and infusions, dried fruits, syrups, and fruit-based products [14,15]. Dietary supplements containing white mulberry leaf extracts have been present on the market for over a decade, mainly as capsules or tablets targeted at individuals with obesity, hyperglycemia, and diabetes [16]. Despite their widespread commercial availability, mulberry-based supplements often differ substantially in extraction method, phytochemical profile, and degree of standardization (e.g., DNJ or total phenolic content), which may significantly influence their biological activity and reproducibility across studies [17].

Emerging evidence indicates that one of the key biological effects of mulberry-derived materials is modulation of the gut microbiota. Given the important role of the gut microbiota in energy absorption, inflammation regulation, and lipid and glucose metabolism, this interaction is highly relevant to the pathogenesis of metabolic disorders [18,19]. Microbiota-derived metabolites such as butyrate are known to support intestinal epithelial integrity and exert anti-inflammatory effects, whereas dysbiosis-associated lipopolysaccharide (LPS) translocation may contribute to low-grade metabolic endotoxemia and insulin resistance. Alterations in the composition and function of the gut microbiota may contribute to the development of obesity, insulin resistance, type 2 diabetes mellitus, and other metabolic disorders, highlighting the importance of further research into gut microbiota and its potential therapeutic applications for these conditions [20,21]. Experimental studies with mulberry products report shifts in gut microbial composition, including enrichment of members of the former genus *Lactobacillus* (now distributed across genera such as *Lactiplantibacillus*, *Lacticaseibacillus*, *Levilactobacillus*, and others) and *Bifidobacterium*, alongside an increase in other short-chain fatty acid (SCFA)-associated taxa (*Akkermansia*, and SCFA-producing Lachnospiraceae) and a reduction in dysbiosis-associated bacteria [22,23,24]. Notably, the magnitude and direction of these effects depend on the chemical profile of the material administered, which in turn is determined by species, plant part, and processing approach. This review, therefore, examines the effects of mulberry on gut microbiota and gut functionality with explicit consideration of the raw material and technological context, addressing a gap in the literature where findings are often discussed without sufficient attention to the form and origin of the material used.

## 2. Literature Search Strategy

This narrative review was based on a structured literature search conducted in PubMed, Scopus, and Web of Science databases. Searches were performed using terms related to mulberry species and plant materials (“mulberry”, “*Morus alba*”, “*Morus nigra*”, “mulberry leaf”, “mulberry fruit”), combined with topic-specific keywords describing (i) phytochemical composition and bioactive compounds (“phytochemical composition”, “polyphenols”, “anthocyanins”, “polysaccharides”, “DNJ”, “alkaloids”), (ii) processing methods (“processing”, “drying”, “freeze-drying”, “fermentation”, “extraction”), and (iii) gut microbiota and intestinal function (“gut microbiota”, “SCFA”, “intestinal barrier”, “gut health”).

Publications were screened based on title, abstract, and full-text content. Studies were considered eligible if they investigated mulberry leaves, fruits, or derived products in relation to phytochemical composition, processing-induced changes, biological activity, gut microbiota, intestinal barrier function, or other aspects of gut functionality. Original research articles and relevant review papers published in English were considered. Publications unrelated to *Morus* species, studies focusing on plant parts outside the scope of this review, conference abstracts, editorials, commentaries, and articles without accessible full text were excluded.

## 3. Main Bioactive Components of Mulberry Leaves and Fruits

### 3.1. Chemical Composition of Mulberry Leaves

Leaves of both *M. alba* and *M. nigra* represent a nutrient-dense plant material characterized by high protein and fiber contents together with substantial mineral accumulation. Fresh *M. alba* leaves typically contain 71–77% moisture, whereas drying concentrates nutrients, resulting in protein levels of approximately 15–31% of dry weight (DW) and fiber fractions reaching 28–37% DW when expressed as neutral detergent fiber (NDF) [25]. Across studies, protein contents generally range from 13% to 31% DW, while lipid concentrations remain relatively low (2–7% DW) [25,26,27,28,29].

Considerable variation in proximate composition has been reported among genotypes and species. For example, six *M. alba* genotypes cultivated under the same conditions in India differed nearly two-fold in protein content (15.3–30.9% DW) and three-fold in carbohydrate content (9.7–29.6% DW), highlighting a strong genotypic effect [25]. In a direct comparison conducted in Pakistan, *M. nigra* leaves contained more crude fiber than *M. alba* (12.3 vs. 10.1% DW), whereas protein, lipid, and ash contents were broadly comparable between species [27]. Available data indicate that soluble sugars constitute only a minor fraction of total leaf carbohydrates, with glucose and fructose together reaching approximately 0.6 g/100 g of fresh weight (FW) in *M. alba* leaves [30].

Mulberry leaves are also rich in mineral constituents, particularly calcium, potassium, and magnesium [25,26,31]. Substantial variation in mineral composition has been observed among cultivars and geographical origins. In *M. alba*, calcium concentrations ranged from 787 to 2227 mg/100 g DW among six Indian genotypes [25], while Spanish clones differed markedly in potassium (1200–3900 mg/100 g DW) and magnesium contents (500–1400 mg/100 g DW) [26]. Comparative data from Pakistan suggest species-related differences in mineral accumulation, with *M. nigra* containing higher levels of calcium (2054 vs. 1347 mg/100 g DW) and potassium (2072 vs. 1355 mg/100 g DW), whereas *M. alba* exhibited higher concentrations of iron (29.0 vs. 19.1 mg/100 g DW) and zinc (5.09 vs. 1.61 mg/100 g DW) [31]. Overall, the available evidence indicates that both genotype and growing environment contribute substantially to the compositional variability of mulberry leaves.

### 3.2. Chemical Composition of Mulberry Fruits

Mulberry fruits of both *M. alba* and *M. nigra* are highly hydrated tissues, typically containing 72–82% water, with carbohydrates representing the predominant macronutrient fraction [32,33,34]. Compared with leaves, fruit carbohydrates are largely present as soluble sugars, predominantly glucose and fructose, whereas sucrose occurs at lower concentrations [35]. Protein, lipid, and mineral contents are comparatively lower, although fruits also provide nutritionally relevant amounts of dietary fiber [32,33,34,36,37,38,39,40,41].

Available data indicate that compositional differences between *M. alba* and *M. nigra* fruits are generally less pronounced than the variability observed among cultivars and geographical regions. In Egypt, both species exhibited very similar protein (7.35–7.40%), fiber (4.2–4.4%), and ash contents (4.2–4.5%), while *M. alba* contained slightly more lipids than *M. nigra* (10.18 vs. 8.83%) [34]. Likewise, fruits collected in Pakistan showed comparable carbohydrate and ash contents between species, although *M. nigra* contained considerably more fiber than *M. alba* (11.75 vs. 1.47 g/100 g FW) [32]. In contrast, substantial regional variability was observed within *M. nigra*. Fruits originating from Northern India contained 4.69% protein, 5.52% fiber, and 1.05% lipids [33], whereas Egyptian material contained 7.35% protein, 4.20% fiber, and 8.83% lipids [34]. Genotypic effects were also evident in *M. alba*, with five Philippine cultivars differing in moisture (22.6–44.9%), carbohydrate (66.8–74.4 g/100 g FW), and ash contents (0.88–1.14 g/100 g FW), depending on cultivar and growing season [38]. Similarly, four Korean cultivars showed substantial variation in protein (8.3–11.9% DW), fiber (7.5–10.8% DW), and lipid contents (4.3–5.5% DW) [42].

Structural carbohydrates appear less abundant in fruits than in leaves and remain relatively poorly characterized. Nevertheless, pectin was detected in *M. nigra* fruits (0.76 g/100 g FW) but not in *M. alba* under identical analytical conditions, suggesting potential species-related differences in polysaccharide composition [43].

Fruit mineral composition is likewise highly variable and appears strongly influenced by geographical origin [44]. Potassium consistently represents the dominant macroelement, followed by calcium and magnesium [32,36,39]. However, interspecies trends are inconsistent. In Pakistan, *M. alba* fruits contained higher potassium (1731 vs. 1270 mg/100 g FW) and calcium (576 vs. 470 mg/100 g FW) concentrations than *M. nigra*, whereas *M. nigra* exhibited higher iron (77.6 vs. 73.0 mg/100 g FW) and zinc contents (59.2 vs. 50.2 mg/100 g FW) [32]. In contrast, fruits grown in Bahrain contained substantially higher concentrations of calcium, magnesium, sodium, and zinc in *M. nigra* than in *M. alba* [36]. These findings indicate that regional and cultivar-dependent effects may contribute more strongly to fruit mineral composition than species differences alone.

### 3.3. Vitamins in Morus alba and Morus nigra Leaves and Fruits

Vitamins constitute an important component of the nutritional profile of mulberry leaves and fruits, with the most consistently reported compounds including vitamin C (ascorbic acid), provitamin A (carotenoids, particularly β-carotene), and selected B-group vitamins. Their concentrations vary substantially depending on plant organ, processing (fresh vs. dried), genotype, and environmental conditions [3,25,45,46,47,48].

#### 3.3.1. Vitamins in Mulberry Leaves

Mulberry leaves, particularly those of *M. alba*, are recognized as a rich source of vitamin C and carotenoids. Fresh leaves have been reported to contain approximately 160–280 mg/100 g of ascorbic acid and 10,000–14,688 µg/100 g of β-carotene, while in dried leaf powder these values decrease to approximately 100–200 mg/100 g and 8438–13,125 µg/100 g, respectively [25]. However, markedly lower concentrations of ascorbic acid have also been reported in shade dried mulberry leaves from Syrian cultivars, ranging from approximately 1.7 to 3.8 mg/100 g DW depending on species [47]. In addition to these dominant vitamins, the presence of B-group vitamins such as riboflavin (B2) and niacin (B3) has been confirmed, although their concentrations are considerably lower [45]. In selected *M. alba* genotypes, riboflavin and niacin have been reported at levels up to 0.49 mg/g and 0.78 mg/g dry weight, respectively, while ascorbic acid reached up to 2.39 mg/g in the same datasets [27]. Across studies, β-carotene consistently appears as the predominant vitamin in mulberry leaves, whereas niacin is typically present at the lowest levels [45,46,49].

#### 3.3.2. Vitamins in Mulberry Fruits

In contrast to leaves, mulberry fruits contain lower but still nutritionally relevant concentrations of vitamins, with vitamin C being the most consistently quantified compound [32,33,35,50]. Across multiple studies, vitamin C levels in *M. alba* and *M. nigra* fruits typically range from approximately 10 to 30 mg/100 g fresh weight [3,32,35,47]. For instance, fruits of *M. alba* and *M. nigra* have been reported to contain 15.2–17.0 mg/100 g vitamin C [32], while Turkish cultivars of white, black, and red mulberries exhibited values ranging from 11.3 to 24.4 mg/100 g [35]. Higher concentrations have been reported in certain regional samples, such as black mulberry fruits from Himachal Pradesh (31.22 mg/100 g), indicating substantial variability linked to genotype and environmental conditions [33]. In addition to vitamin C, mulberry fruits may contain smaller amounts of other vitamins, including carotenoids, vitamin E, thiamine (B1), and folate derivatives, although quantitative data for these compounds are less consistently reported [3,14,47,51]. For example, vitamin E has been detected at levels around 0.98 mg/100 g in *M. nigra* fruits [51]. Comparative analyses suggest that *M. nigra* fruits often exhibit higher vitamin C content than *M. alba*, although this trend is not consistent across all regions and cultivars [3,51].

### 3.4. Bioactive Compounds in Mulberry Leaves

**Figure 2:**
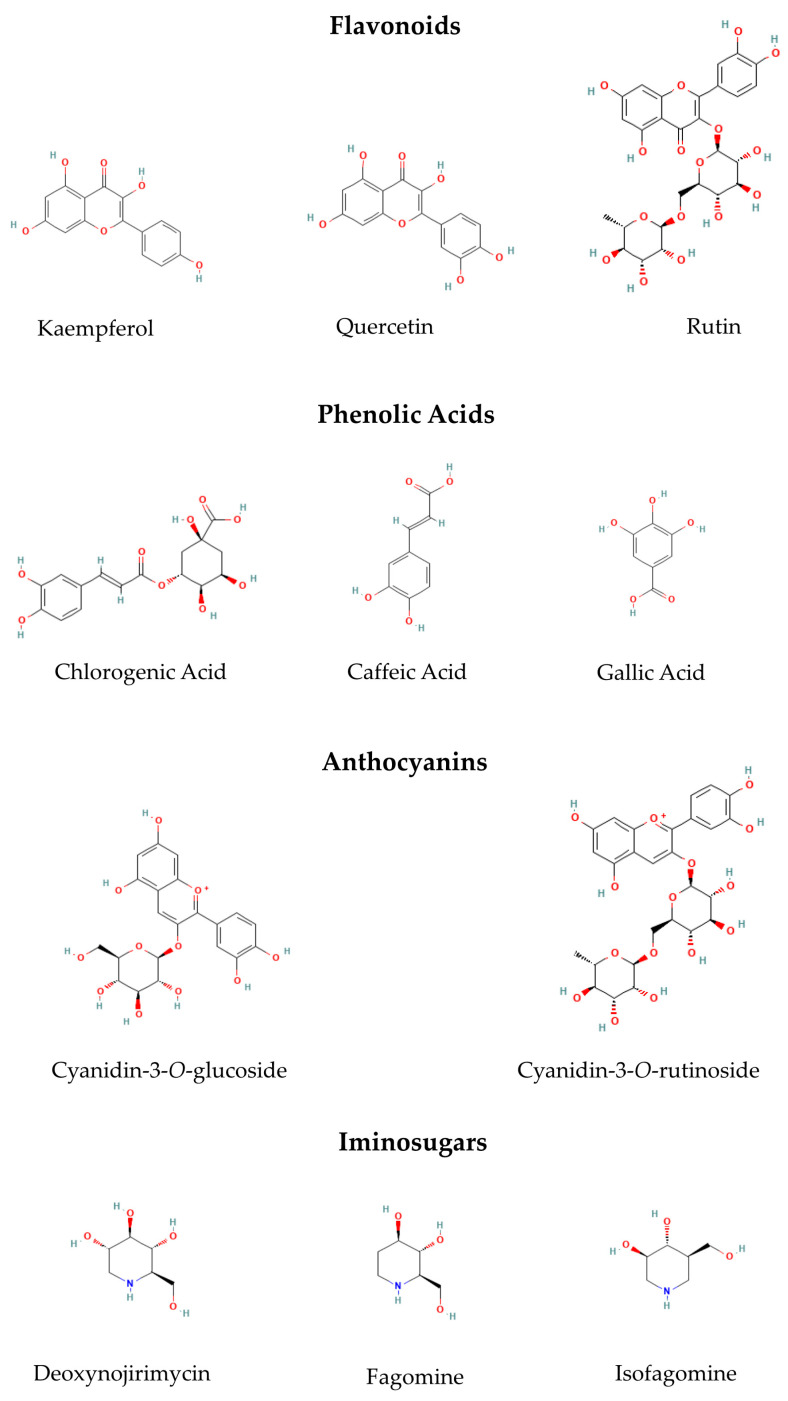
Main chemical structures of representative phytochemicals identified in the leaves and fruits of *M. alba* and *M. nigra* [https://pubchem.ncbi.nlm.nih.gov/ (accessed on 26 June 2026)]. Flavonols (quercetin and kaempferol) and their glycosides (e.g., rutin) possess a characteristic C6–C3–C6 flavonoid backbone; glycosylation increases their polarity and may influence bioavailability. Phenolic acids (chlorogenic, caffeic, and gallic acids) are smaller phenolic compounds containing one aromatic ring substituted with hydroxyl and carboxyl groups, and contribute to antioxidant and metabolic effects. Anthocyanins (cyanidin glycosides) are structurally related to flavonoids but contain a positively charged flavylium core responsible for the red–purple pigmentation of mulberry fruits and their antioxidant activity. In contrast, iminosugars (1-deoxynojirimycin—DNJ, fagomine—FAG, and isofagomine) are nitrogen-containing sugar analogues that structurally resemble monosaccharides and act as α-glucosidase inhibitors, thereby contributing to postprandial glucose regulation [54].

Mulberry leaves are characterized by a complex and diverse phytochemical profile, including flavonoids, alkaloids, phenolic acids, and polysaccharides (Figure 2). Most phytochemical investigations have historically focused on *M. alba* leaves, whereas data for *M. nigra* were more limited. However, recent advanced profiling studies have substantially expanded the phytochemical characterization of both white and black mulberry leaves [52,53].

Flavonoids constitute one of the key groups of bioactive compounds present in *M. alba* leaves and are largely responsible for their antioxidant properties. Phytochemical studies have identified both newly discovered and previously described flavonol derivatives. Compounds reported in recent phytochemical investigations include kaempferol-7-*O*-glucoside, quercetin-3-*O*-rhamnoside-7-*O*-glucoside, and quercetin-3-*O*-β-glucoside-7-*O*-α-rhamnoside. Among the known flavonoids detected in white mulberry leaves are rutin, quercetin-3,7-di-*O*-β-*D*-glucopyranoside, quercetin-3-*O*-glucoside, quercetin-3-*O*-(6-malonyl)-β-*D*-glucopyranoside, kaempferol-3-*O*-glucopyranosyl-(1→6)-β-*D*-glucopyranoside, and kaempferol-3-*O*-(6-malonyl)glucoside [26,44]. Additionally, flavan derivatives (moracinflavan A–G) and compounds belonging to the 2-arylbenzofuran group (moracinfurol A and B), which are also classified broadly as polyphenols, have been isolated from the leaves of *M. alba* [55,56]. Among the major bioactive constituents of *M. alba* leaves, rutin, apigenin, and quercetin exhibited the highest biological activity, highlighting the significant role of flavonoids as the predominant class of phenolic active compounds in this plant [53].

In phytochemical studies, seven major alkaloids have been isolated and characterized from white mulberry leaves, including iminosugars: DNJ, 1,N-dimethyl-1-deoxynojirimycin, 2-O-α-*D*-galactosyl-1-deoxynojirimycin (Gal-DNJ), fagomine (FAG), 1,4-dideoxy-1,4-imino-*D*-arabinitol, 1,4-dideoxy-1,4-imino-(2-*O*-β-*D*-glucopyranosyl)-*D*-arabinitol, as well as 1α,2β,3α,4β-tetrahydroxy-demetropine and nortropanolines [57]. DNJ is particularly noteworthy due to its strong α-glucosidase inhibitory activity, which underlies the well-documented antidiabetic potential of mulberry leaves. Among these compounds, DNJ, FAG, and Gal-DNJ constitute more than 80% of the total alkaloid fraction in *M. alba* leaves [57,58]. Other structurally related iminosugars, including isofagomine and 4-*O*-β-*D*-glucopyranosylfagomine (Glu-FAG) have also been identified in mulberry leaves [59]. DNJ is regarded as the most typical mulberry alkaloid and a potent α-glucosidase inhibitor, and serves as a key active marker underlying the antidiabetic activity of mulberry leaf preparations [60]. Analyses of dry leaves representing 58 *M. alba* varieties revealed DNJ concentrations ranging from 0.13 to 1.46 mg/g DW, although substantial variation was observed depending on leaf maturity and harvest conditions [61]. Young leaves generally contained higher amounts of DNJ (0.10–1.61 mg/g) than mature leaves (0.47–0.96 mg/g) [62]. Seasonal fluctuations in alkaloid composition have also been reported. Analyses performed using hydrophilic interaction liquid chromatography coupled with tandem mass spectrometry (HILIC-MS/MS) demonstrated that the highest DNJ concentration occurs during the summer months (June–July), the maximum fagomine content is observed in spring (April–May), whereas Gal-DNJ and Glu-FAG reach their peak levels in autumn (September–October) [63]. The seasonal variations in iminosugars are strongly associated with changes in growing temperature [58]. Data on DNJ content in *M. nigra* leaves are considerably more limited. However, the available evidence suggests that DNJ concentrations may be comparable between *M. alba* and *M. nigra*, with reported levels of 0.103% and 0.102% DW, respectively [64]. Owing to its abundance, biological activity, and relatively well-characterized occurrence in mulberry leaves, DNJ is widely used as a marker compound for quality assessment and standardization of mulberry leaf-derived products [65].

In addition to flavonoids, other classes of polyphenols have been identified, including benzofurans, phenolic acids, coumarins, stilbenes, and caffeoylquinic acid derivatives, as well as chlorogenic acid and oxyresveratrol, which further contribute to the bioactivity of the *M. alba* leaves. To date, more than 140 polyphenolic compounds have been identified, with flavonoids and their derivatives representing the predominant fraction [66].

A comparable spectrum of phenolic compounds has been reported in *M. nigra* leaves, including rutin, quercetin, and kaempferol derivatives [67,68]. These data confirm that black mulberry leaves are polyphenol-rich organs dominated by flavonol glycosides and chlorogenic-type phenolic acids, with DNJ alkaloids as important complementary metabolites. Recent HPLC-ESI-QTOF-MS profiling studies have substantially expanded the phytochemical characterization of *M. nigra* leaves, revealing not only phenolic acids and flavonoids, but also lignans, glycolipids, capsinoid derivatives, and oxidized fatty acid derivatives. Several compounds, including kuwanon C, syringaresinol, dihydrocapsiate isomers, and gingerglycolipid A, were reported in *M. nigra* leaves for the first time [69].

Mulberry leaves are also a source of bioactive heteropolysaccharides, which typically account for approximately 7–24% of leaf dry weight depending on the cultivar and extraction method [70]. Their monosaccharide composition typically includes arabinose, galactose, glucose, rhamnose, xylose, mannose, and uronic acids, although the relative abundance of individual sugars varies among fractions and plant materials. Structural analyses indicate that many mulberry leaf polysaccharides (MLPs) possess pectic and arabinogalactan-like features. For example, the well-characterized mucilaginous polysaccharide fraction isolated from white mulberry leaves was identified as a rhamnogalacturonan rich in galacturonic and glucuronic acid residues [71]. Beyond their antioxidant, immunomodulatory, and hypoglycemic activities, the MLPs have attracted growing interest as potential prebiotic compounds capable of interacting with the gut microbiota.

In addition to polyphenols, alkaloids, and polysaccharides, mulberry leaves also contain γ-aminobutyric acid (GABA), a non-protein amino acid with recognized neuroactive and physiological functions. Basal GABA concentrations in mulberry leaves have been reported at approximately 1.17 mg/g DW, although post-harvest stress treatments such as anaerobic incubation and cold shock may increase its content to 3.0–3.6 mg/g DW [72]. Notably, substantial increases in GABA concentrations have also been reported following microbial fermentation of mulberry leaves, indicating that this compound is highly responsive to processing conditions [73,74]. Beyond its role in the nervous system, GABA is increasingly recognized as a microbiota-related metabolite involved in host–microbe signaling pathways [75].

### 3.5. Bioactive Compounds in Mulberry Fruits

*Morus* fruits are rich in bioactive compounds (Figure 2), all of which play critical roles in determining antioxidant capacity and associated health benefits [76]. The one of the phytochemical class of mulberries are flavonoids and phenolic acids [16]. Their content in these fruits is higher than in other berry species like blueberry, strawberry, blackberry, and raspberry. Previous studies have identified at least 17 individual phenolic compounds in *M. alba* fruits, including cinnamic acid derivatives, flavonols, and benzoic acid derivatives, confirming their quantitative significance. In addition, volatile compounds, such as aldehydes, esters, ketones, benzene derivatives, and terpenes, have been detected, contributing to the fruit’s characteristic aroma profile and potential bioactivity [40,77]. Anthocyanins are the major pigments and key bioactive compounds in mulberry fruits, particularly in *M. nigra*, although their content varies significantly among cultivars. Anthocyanin content in black mulberry fruits can reach up to 1.88 mg/g [40]. Notably, high anthocyanin concentrations are not restricted to *M. nigra*, as dark-colored cultivars of *M. alba* have been reported to contain up to 5.7 mg/g fresh weight of anthocyanins [50,78]. The most abundant anthocyanin in mulberry is cyanidin-3-*O*-glucoside (Cya-3-Glu), representing 53.94–78.23% of the total anthocyanins; cyanidin-3-*O*-rutinoside accounts for 19–43.83%, and pelargonidin-3-*O*-glucoside is measured in a proportion close to 5% [79]. These pigments are water-soluble and exhibit significant bioactivity, although their stability depends on environmental factors such as pH, temperature, and light. In *M. alba*, purple-colored fruits were found to contain the highest anthocyanin levels and exhibited the strongest antioxidant and anti-tyrosinase activities among the tested fruit color variants. However, prolonged exposure of the extract to heat (70 °C for 10 h) and light significantly reduced anthocyanin and ascorbic acid contents, accompanied by a decline in biological activity, highlighting the susceptibility of these compounds to processing conditions [78].

The total alkaloid content of mulberry fruits, determined gravimetrically, has been reported at approximately 660 mg/100 g FW in *M alba* and 630 mg/100 g FW in *M. nigra* [32]. In mulberry fruits, DNJ is present at considerably lower concentrations than those reported for leaves. Analysis of 16 white mulberry fruit varieties revealed DNJ levels ranging from 0.57 to 13.2 mg/100 g FW. Moreover, DNJ was found predominantly in young fruits and declined during fruit maturation [80]. It has been shown that mulberry fruits contain several nortropane alkaloids and pyrrole alkaloids such as morroles and their derivatives [81,82].

Moreover, in five white mulberry varieties grown in a tropical region, GC–MS identified 100 volatiles, including 11 terpene alcohols such as linalool, leaf alcohol, n-hexanol, benzyl alcohol, and α-terpineol; the proportion of terpene alcohols differed among varieties, contributing to distinct fruit aromas [83]. Beyond phenolic compounds and alkaloids, mulberry fruits are also a source of biologically active polysaccharides, which have been reported to exhibit antioxidant, immunomodulatory, and anti-inflammatory properties [40]. Stilbenes and other flavonoids, including catechins (e.g., epigallocatechin, gallocatechin), isorhamnetin derivatives, kaempferol derivatives, cyanidin derivatives, and resveratrol derivatives, have also been identified in *Morus* species and further contribute to their overall antioxidant potential [6,16,84]. Tropical conditions (high temperature and humidity) increased the main nutritional components of mulberry fruits relative to earlier ripening stages, although specific molecular mechanisms remain unclear [78,85]. This suggests that warmer, high-radiation environments may enhance some aroma fractions.

## 4. Influence of Processing and Extraction on Phytochemical Composition

Mulberry leaves and fruits are widely utilized in a variety of processed food products, including teas, juices, syrups, jams, dried fruits, wines, vinegars, and fermented beverages. These products have been extensively characterized with respect to their sugar composition, vitamin C content, phenolic profile, anthocyanins, antioxidant capacity, and volatile compounds. Thermal treatments often reduce anthocyanin and phenolic contents, whereas fermentation can decrease vitamin C and sugar levels and generate new metabolites with potentially different bioavailability and biological effects [86,87]. While traditionally consumed mulberry foods contain complex mixtures of phytochemicals within their natural matrix, experimental studies frequently employ concentrated extracts or purified fractions enriched in specific groups of bioactive compounds. Consequently, both the composition of the preparation and the method of its production should be considered when evaluating microbiota-related effects and comparing results across studies. Additional evidence suggests that even simple processing methods used in the preparation of mulberry-based beverages may substantially influence phytochemical composition. Comparative analyses of *M. alba* and *M. nigra* leaves showed that infusions, decoctions, and tinctures differed markedly in their phenolic profiles and antioxidant activity, indicating that the mode of preparation can affect the recovery of bioactive compounds and the functional properties of the final product [53].

### 4.1. Pretreatment of Plant Material

Pretreatment of plant material aims to stabilize the raw matrix prior to extraction or further processing. It limits enzymatic degradation and facilitates the release of intracellular phytochemicals. For mulberry leaves and fruits, common steps include washing, drying, grinding with particle-size standardization, and storage under controlled conditions [88].

Drying is a key operation, reducing moisture and microbial growth while affecting phytochemical stability. Techniques include air drying, hot-air/oven drying, and freeze-drying [89]. Each of these methods may influence the phytochemical composition of the plant material differently. For example, freeze-drying often preserves structural integrity and antioxidant activity of polysaccharides better than hot-air drying, whereas moderate-temperature air-drying can retain phenolics with limited degradation [90]. Mechanical size reduction by grinding/pulverization increases surface area, improves solvent penetration and typically involves sieving to standardize particle size and extraction reproducibility [91]. Additional pretreatments such as roasting, fermentation, or withering are used particularly for mulberry leaf tea and functional ingredients. These processes can induce biochemical transformations that modify the phenolic profile and antioxidant activity [88,92].

It should be noted that direct comparisons among studies investigating mulberry leaves and fruits are often complicated by substantial methodological heterogeneity. Dif-ferences in pretreatment conditions, including drying temperature and duration, particle size, storage conditions, fermentation procedures, and roasting intensity, may significant-ly influence the stability and extractability of phytochemicals. Furthermore, variations in extraction protocols (solvent type, solvent concentration, extraction time, temperature, and solid-to-solvent ratio), analytical techniques used for compound quantification, and experimental models employed for biological activity assessment may contribute to discrepancies among reported results. Therefore, differences in phytochemical composition and bioactivity observed across studies should be interpreted with caution, taking into account the specific methodological approaches applied.

#### 4.1.1. Mulberry Leaves

Freshly harvested mulberry leaves are usually washed, dried under controlled conditions, ground, sieved and stored to stabilize the material and enhance extraction. A frequently used protocol dries *M. alba* leaves in a convection dryer at 60 ± 1 °C for 6–8 h, followed by grinding and sieving to 0.08–0.8 mm, and storage in airtight containers at ~4 °C to prevent oxidation of phenolics [91]. Similar approaches use dried, powdered leaves extracted with hydroorganic solvents such as 65% acetone or 65% ethanol at elevated temperature, followed by filtration, solvent removal, and freeze-drying to obtain stable extracts [93]. Alternative drying conditions include oven-drying at 45 °C for ~48 h, with subsequent pulverization and storage at −20 °C; lower temperatures over longer times help preserve heat-sensitive phenolics and flavonoids [88].

For mulberry leaf tea, additional processing such as roasting (e.g., ~220 °C), rolling, and non-fermented or partially/fully fermented schemes (withering, rolling, controlled fermentation, then drying) are applied. These treatments alter the phytochemical profile, including concentrations and stability of rutin and other flavonoids [88,92]. Drying methods also influence polysaccharides and antioxidant properties. For example, one study reported that freeze-drying yielded approximately 28% more polysaccharides and higher antioxidant activity than hot-air drying [90]. However, the magnitude of these differences may vary, depending on factors such as cultivar, leaf maturity, drying conditions, extraction procedures, and analytical methods used for polysaccharide determination, which limits direct comparison among studies. Temperature control during drying is another critical factor affecting the stability of phenolic compounds. Research has shown that drying mulberry leaves at temperatures of 60 °C or below preserves antioxidant activity and the levels of key polyphenols such as quercetin glycosides and chlorogenic acid, whereas higher temperatures (≥70 °C) may lead to significant degradation of these compounds [89].

In some phytochemical studies, leaves of both *M. alba* and *M. nigra* have also been directly pulverized using laboratory grinders, producing homogenized plant material suitable for further chemical analyses. The powdered samples are typically stored in dry, cool conditions and protected from light in order to prevent degradation of sensitive phytochemicals prior to extraction or chromatographic characterization [53]. Overall, washing, drying, grinding, and optional thermal or fermentation steps strongly determine the composition and stability of mulberry leaf preparations, affecting yields of flavonoids, phenolic acids, and polysaccharides and thus their biological properties.

#### 4.1.2. Mulberry Fruits

Mulberry fruits undergo pretreatment to stabilize the highly perishable, water-rich tissue, prevent degradation of sensitive metabolites and facilitate extraction. Ripe fruits are typically selected, washed and dried before grinding. For example, fruits collected in India were washed, shade-dried at room temperature for 4–5 days, ground to fine powder and stored in airtight containers at ~4 °C [94]. Drying profoundly affects fruit phytochemicals. Compared methods include hot-air drying, vacuum drying, and freeze-drying. Freeze-drying is generally most effective in preserving anthocyanins, flavonoids, vitamin C, and antioxidant activity [95,96]. Simpler shade- or low-temperature drying (≤55–65 °C) can also retain phenolics and antioxidant properties; lower temperatures tend to preserve bioactives better than high-temperature treatments [97]. Another commonly used method involves hot-air drying of mulberry fruits at temperatures ranging from 50 to 70 °C until constant weight is achieved. Drying temperature is a critical factor influencing the stability of bioactive compounds, as moderate temperatures (around 50–60 °C) generally allow better retention of phenolic compounds and antioxidant activity compared with higher temperatures. However, increasing temperature may lead to degradation of anthocyanins and other thermolabile constituents. Dried fruits are then ground and stored at low temperature (e.g., 4 °C) to limit further degradation [95,96]. Freeze-drying involves freezing followed by ice sublimation under reduced pressure, minimizing thermal damage [98]. It improves retention of anthocyanins such as cyanidin-3-glucoside and cyanidin-3-rutinoside relative to hot-air drying and is associated with higher antioxidant activity and better preservation of color and vitamin C [99,100].

Overall, pretreatment strategies strongly affect the retention and extractability of bioactive compounds in mulberry fruits, thereby influencing the phytochemical composition and biological properties of the resulting extracts.

### 4.2. Extraction as the Main Method of Isolating Phytochemicals

The extraction of bioactive compounds from plant material largely depends on solvent choice, as different compound classes show distinct solubility according to extraction medium polarity. Common solvents include water, alcohols (methanol, ethanol), acetone, and their mixtures [90,91]. Highly polar solvents, such as water and hydroalcoholic mixtures, are particularly effective for extracting polyphenols, flavonoids, and anthocyanins, which represent a major fraction of mulberry bioactive metabolites [50].

Besides solvent polarity, extraction efficiency is strongly influenced by the applied technique. Conventional methods, such as maceration and Soxhlet extraction, have long been used, but modern strategies increasingly rely on ultrasound assisted extraction (UAE), microwave assisted extraction (MAE), and pressurized liquid extraction (PLE), which enhance efficiency, shorten extraction time and reduce solvent consumption while maintaining high recovery of target compounds [101].

Such approaches have been applied to *M. alba* leaves, where UAE was optimized for the recovery of phenolic compounds using HPLC-MS evaluation of extraction efficiency and antioxidant activity. Extraction yield was strongly influenced by ethanol concentration, extraction time, and solvent-to-solid ratio, while UAE provided phenolic recovery comparable to PLE with reduced extraction cost and processing time [102]. More recent studies have additionally employed PLE and supercritical fluid extraction (SFE) for *M. nigra* leaves. Comparative analyses demonstrated that PLE and maceration provided comparable recovery of phytochemicals, whereas PLE substantially reduced extraction time and improved process efficiency. HPLC-ESI-QTOF-MS analyses identified phenolic acids, flavonoids, and fatty acid derivatives as major components of the obtained extracts and additionally revealed 13 compounds not previously reported in *M. nigra* leaves [69]. Importantly, extract composition depended not only on extraction technique itself but also on solvent combinations and extraction temperature, highlighting the strong interaction between extraction parameters and phytochemical selectivity.

Beyond technique and solvent, phytochemical recovery is influenced by plant-related factors such as species, plant part, maturity, cultivation conditions, and post-harvest processing. Studies show that total phenolic content (TPC) and total flavonoid content (TFC) vary greatly with ripening stage and environment, affecting antioxidant capacity [40,50]. Comparative studies of 70% methanol, 60% ethanol, and 65% acetone have shown that solvents of similar, moderate polarity can differ markedly in selectivity toward specific phytochemical groups, generating distinct extract profiles. Operational parameters—including temperature, time, solvent-to-solid ratio, and particle size—also affect extraction yield; their optimization is crucial to maximize recovery while limiting degradation of thermolabile metabolites such as anthocyanins and some flavonoids [103,104].

In mulberry matrices, extraction procedures are usually designed to recover phenolics such as chlorogenic acid, rutin, quercetin derivatives, and anthocyanins, major contributors to the antioxidant activity of fruits and leaves. Crude extracts are often further purified by solvent fractionation or chromatographic separation to simplify the complex phytochemical mixture and facilitate identification of individual bioactive compounds [42,96].

#### 4.2.1. TPC of Mulberry Fruits and Leaves

Phenolic extraction from mulberry fruits and leaves is strongly governed by solvent composition, particularly polarity, which determines solubility and recovery of diverse phenolic structures. Unlike polysaccharides, mainly obtained with water-based systems, phenolics are more efficiently extracted with aqueous organic solvents such as ethanol, methanol, or acetone. Studies consistently show that intermediate polarity mixtures (typically 50–70% *v*/*v*) give the highest TPC by promoting cell wall penetration and solubilization of bound phenolics. In Piechocka et al., among 70% methanol, 60% ethanol, and 65% acetone, 65% acetone produced the highest TPC at all fruit maturity stages, with values ranging from 4.96 to 9.26 mg gallic acid equivalent (GAE)/g DW [105]. Lower TPC values were obtained for 60% (*v*/*v*) ethanol (1.71–3.78 mg GAE/g DW) and 70% (*v*/*v*) methanol (1.37–2.52 mg GAE/g DW). The TPC obtained with 65% acetone was approximately 58.96% higher than that achieved with 60% ethanol and 70.79% higher than with 70% methanol [104,106]. Thus, solvent selection is a key determinant of phenolic extraction efficiency and must be carefully controlled when comparing phytochemical profiles across studies.

Similar solvent-dependent trends in total phenolic content have also been reported for both *M. alba* and *M. nigra* in independent studies. For instance, in *M. alba* fruits extracted with aqueous ethanol systems, TPC values typically ranged between 7.0 and 13.6 mg GAE/g DW, depending on ethanol concentration and fruit maturity stage, whereas methanolic extracts yielded comparatively lower or similar values under analogous conditions [42]. In white mulberry leaves, ethanolic and methanolic extracts contain 8.76–20.26 mg GAE/g DW, with younger leaves generally richer in phenolics than mature ones. Comparisons of solvent systems show that aqueous acetone often affords superior phenolic recovery versus pure or highly aqueous alcohols, particularly when phenolics are associated with cell wall or protein matrices [13]. For *M. nigra* fruits, methanolic extracts show TPC values of 7.7–11.2 mg GAE/g DW, while ethanolic extracts give comparable but slightly variable results depending on temperature and solvent concentration [40]. Beyond conventional solvent extraction, supercritical fluid extraction has also been explored for recovery of phenolic compounds from black mulberry fruits. Using supercritical CO_2_ with ethanol as a co-solvent (35 MPa, 45 °C), a polyphenol-rich light-yellow extract was obtained with an extraction yield of 1.9% (*w*/*w*), demonstrating the feasibility of solvent-reduced extraction approaches for the production of concentrated mulberry phenolic preparations [107].

#### 4.2.2. TFC of Mulberry Fruits and Leaves

Flavonoids, as moderately polar compounds, are most effectively extracted using aqueous organic solvents, particularly ethanol and acetone mixtures in the range of 50–70% (*v*/*v*). Experimental studies consistently demonstrate that flavonoid recovery is highly sensitive to solvent system composition, with intermediate-polarity mixtures generally enhancing extraction efficiency; however, reported TFC values vary markedly across studies due to differences in solvent type, aqueous proportion, and extraction protocols, often leading to substantial discrepancies even when similar plant materials are analyzed [106,108]. These variations highlight that, in addition to extraction parameters, biological factors such as genotype and fruit maturity significantly influence flavonoid accumulation and measured TFC values [42,85,106].

A similar solvent- and maturity-dependent pattern was observed for total flavonoid content (TFC). Fully ripe fruits exhibited 21.72% higher TFC values, ranging from 25.03 to 40.60 mg quercetin equivalents (QUE)/g DW, compared with red mature fruits (15.00–35.11 mg QUE/g DW), while 65% (*v*/*v*) acetone extracts yielded the highest flavonoid levels at both maturity stages [106]. These findings are consistent with previous reports demonstrating an increase in total flavonoid content during mulberry fruit ripening, reaching 0.1–0.4 g/100 g DW in mature fruits [42].

Additional studies confirm the high flavonoid abundance in mulberry matrices and indicate that their concentration strongly depends on the plant organ analyzed. In a recent comparative analysis of different parts of *M. alba*, flavonoids such as quercetin, isoquercetin, kaempferol, and epicatechin were identified using HPLC. The content of quercetin ranged from 36.2 to 59.3 mg/100 g, while isoquercetin ranged from 15.4 to 30.4 mg/100 g. Among the analyzed tissues, fruits contained the highest flavonoid levels, followed by roots, shoots, and leaves, indicating substantial variability in flavonoid distribution within the plant [48]. Similarly, quantitative analyses of mulberry fruits have reported total flavonoid contents ranging from approximately 29 to 276 mg QE/100 g fresh weight depending on cultivar, geographical origin, and analytical method used. Such variability reflects differences in genotype, environmental conditions, and ripening stage, all of which significantly influence flavonoid biosynthesis in mulberry fruits [79].

#### 4.2.3. Total Anthocyanin Content (TAC) of Mulberry Fruits

In the case of anthocyanins, extraction efficiency is governed not only by solvent polarity but also by solvent acidity and the chemical stability of these pigments. Anthocyanins are highly sensitive to pH, temperature, and solvent composition, and therefore are most effectively extracted using acidified aqueous alcohol systems, particularly ethanol or methanol [106]. Studies have demonstrated that acidification of extraction solvents significantly enhances anthocyanin recovery by stabilizing their flavylium cation form, thereby preventing degradation and structural transformations during extraction and analysis [109].

In comparison to the trends observed for TPC and TFC, the highest TAC values were obtained using 60% (*v*/*v*) ethanol (0.77–7.15 mg Cya-3-Glu/g DW), followed by 70% (*v*/*v*) methanol (0.49–5.84 mg Cya-3-Glu/g DW), whereas 65% (*v*/*v*) acetone yielded the lowest anthocyanin concentrations (0.16–2.34 mg Cya-3-Glu/g DW). The greater efficiency of 60% (*v*/*v*) ethanol and 70% (*v*/*v*) methanol in determining total anthocyanin content (TAC), compared with 65% (*v*/*v*) acetone (by approximately 68% and 20%, respectively), may be attributed to their ability to penetrate plant tissues, disrupt membrane structures, and effectively dissolve and stabilize anthocyanin molecules in solution [104]. In contrast, the lower TAC values obtained for acetone extracts may result from potential interactions between acetone and anthocyanins, leading to the formation of more complex and stable derivatives, such as pyranoanthocyanins. Although these compounds exhibit enhanced chemical stability, they are not fully detectable by the spectrophotometric method commonly used for TAC determination, which may consequently lead to an underestimation of the results [109].

Additional comparative studies have shown that total anthocyanin content and antioxidant capacity differ considerably between mulberry species. In a study comparing white (*M. alba*), red (*M. rubra*), and black (*M. nigra*) mulberries grown in Tunisia, the highest total anthocyanin content was observed in *M. nigra*, reaching 10.05 mg cyanidin-3-glucoside equivalents per 100 g fresh weight. Black mulberry fruits also exhibited the strongest antioxidant activity in the DPPH assay (71.03%), compared with 66.62% for *M. alba*, confirming that darker mulberry species generally possess higher antioxidant potential associated with greater anthocyanin accumulation [43].

#### 4.2.4. Polysaccharides of Mulberry Fruits and Leaves

The extraction efficiency of mulberry leaf and fruit polysaccharides depends strongly on the extraction method and operating conditions. In a comparative study evaluating six extraction procedures for mulberry fruit polysaccharides, extraction yields ranged from 2.10% for water ultrasound extraction to 13.32% for 50% ethanol-assisted ultrasound extraction. The obtained polysaccharide fractions also differed substantially in composition, with total sugar content varying from 64.33% in boiling-water extracts to 92.21% in the 50% ethanol-assisted ultrasound extract. These findings indicate that extraction conditions influence not only polysaccharide recovery but also the chemical purity of the resulting fractions [110]. In addition to the extraction solvent and extraction conditions, downstream purification procedures such as deproteinization, decolorization, ethanol precipitation, dialysis, and fractionation may further modify the composition, molecular-weight distribution, and purity of the resulting polysaccharide preparations. Consequently, biological effects reported for mulberry polysaccharides may reflect not only the extraction process itself but also subsequent processing steps used to obtain the final polysaccharide fraction [70]. The highest polysaccharide values in ethanol extracts could have been the cause of ultrasound waves, which was able to disrupt the cell wall and cell membrane and encourage the leakage of intracellular chemicals. The lowest sugar content found in boiling-water extract may have been caused by the prolonged high temperature and the breaking of glycosidic linkages as well as the higher extraction temperature, which increased the diffusion of impurities and decreased the sugar content.

The most commonly used method for extracting mulberry leaves polysaccharides (MLPs), which has the advantages of simple operation and high cost-effectiveness, is hot-water extraction. Studies have shown that the extraction temperature has the greatest impact on the yield of MLPs, followed by the ratio of material to liquid. The optimal process conditions for material/liquid ratio, extraction temperature, number of extractions, and extraction time are 1:9–1:40, 70 °C−100 °C, 1–3 times, and 60–300 min, respectively. Under these conditions, the highest yield of polysaccharides can reach 15.76% [111]. In another study, temperature was identified as the most significant factor affecting MLPs yield, followed by the water-to-material ratio and extraction time. The yield increased with rising temperature and solvent ratio up to approximately 92–93 °C and 34–40 mL/g, respectively, after which no further improvement was observed. Similarly, extraction times of around 3–3.5 h were found to be optimal, while longer durations led to decreased yields, likely due to partial hydrolysis of polysaccharides. Under optimized conditions (92 °C, 3.5 h, and 34 mL/g), the maximum yield reached 10.0% [112].

Other types of extraction used for extracting MLPs are microwave-assisted extraction, ultrasound-assisted extraction, or enzyme-assisted extraction. To maximize polysaccharide recovery, reduce extraction time and preserve their biological activity, hybrid extraction approaches combining two or more techniques have been developed. Examples include hot-water–enzyme-assisted extraction, microwave–ultrasonic synergistic extraction, and enzymatic hydrolysis–microwave-assisted extraction [111,113,114]. Notably, the combination of hot-water and enzyme-assisted extraction yielded up to 24.04 ± 0.98% crude mulberry leaf polysaccharides, substantially exceeding the yields typically reported for conventional hot-water, microwave- or ultrasound-assisted extraction methods. MLP comprise a structurally diverse group of predominantly acidic heteropolysaccharides containing varying proportions of mannose, rhamnose, arabinose, galactose, glucose, and uronic acids, with several fractions identified as pectic polysaccharides. Considerable variability in molecular weight and monosaccharide composition has been reported among isolated MLP fractions, with molecular weights ranging from only a few kDa to over 2700 kDa depending on the extraction and purification procedures employed [70]. Such differences are likely to contribute to the variability in antioxidant, hypoglycemic, and immunomodulatory activities reported for MLP preparations.

Polysaccharides from mulberry fruits (MFPs) are usually obtained by hot-water extraction, ultrasound-assisted extraction, or combined techniques, with optimized yields in the range of about 3–12% of dry weight, depending on the method and conditions [106,107]. For black mulberry fruit, hot-water extraction at 87 °C for 3 h and a water-to-material ratio of 39 mL/g afforded a crude polysaccharide yield of 11.86% [115]. Ultrasound-assisted extraction of MFPs reached a yield of 3.13 ± 0.07% at 69 °C, 75 min, and a water-to-material ratio of 40.25 mL/g, with progressive enrichment of carbohydrate content (from 58.61% in crude MFP to 81.18% after deproteinization and decolorization) [116]. Fruit polysaccharides are typically acidic heteropolysaccharides composed mainly of mannose, rhamnose, (galacto)uronic acids, galactose, and arabinose, often arranged in highly branched pectic domains together with glucans, xylans, and xyloglucans [115]. Comparative studies of different extraction methods (cold vs. hot water, enzyme-assisted and ultrasonic-assisted hot-water extraction) indicate that techniques such as high-speed shear homogenization-assisted extraction can further increase yields and modify molecular weight, branching degree, particle size, and rheological behavior, while maintaining similar glycosidic linkages [117]. Since the biological activity of MFPs is closely related to their structural characteristics, extraction-induced modifications may affect their functional properties. For example, polysaccharide fractions differing in molecular weight exhibited distinct effects on lipid digestion, with intermediate-molecular-weight fractions displaying the greatest inhibitory activity in vitro [70]. Structural features of MFPs have also been associated with their antioxidant, hypoglycemic, and hypolipidemic activities [115].

#### 4.2.5. Other Phytochemical Composition

The most characteristic alkaloid identified in mulberry leaves is DNJ. A commonly applied approach to alkaloid extraction involves acid-assisted aqueous extraction, in which leaf material is homogenized in diluted hydrochloric acid, followed by ultrasonic treatment to enhance compound release from the plant matrix. After separation of solid residues by centrifugation, repeated extraction of the pellet may be performed to maximize recovery, and the combined extracts are subsequently analyzed by HPLC for quantitative determination of DNJ [118].

An alternative method employs hydroalcoholic extraction, typically using aqueous ethanol under optimized temperature and solvent-to-sample conditions to improve extraction efficiency. Ethanol-based protocols have been reported to provide high recovery of DNJ from dried mulberry leaf powder and may represent an effective option for analytical isolation of this alkaloid [119].

## 5. The Effect of Mulberry Phytochemicals on the Microbiota

Mulberry-derived materials have increasingly been investigated in relation to gut microbiota composition, intestinal barrier function, inflammatory status, and host metabolic homeostasis. The available evidence covers a wide spectrum of preparations, including whole leaves, leaf powders, aqueous, ethanolic, and methanolic extracts, purified polysaccharide fractions, polyphenol-rich fractions, combined polyphenol–polysaccharide preparations, fermented leaves, fermented fruits, fruit pomace, and processed matrices. Most mechanistic and in vivo data concern *M. alba*, particularly leaves and fruits [120,121,122,123,124]. In contrast, direct evidence for *M. nigra* in microbiota-oriented studies remains very limited, and direct comparisons between *M. alba* and *M. nigra* with respect to gut microbiota, intestinal integrity, SCFA production, or inflammation have not yet been reported.

This distinction is important because previous sections of this manuscript describe marked differences in the chemical composition of mulberry leaves and fruits, as well as the influence of extraction solvent on the phytochemical profile of the obtained extracts [120,125,126]. Although such compositional differences could potentially contribute to differences in biological activity, the available evidence is insufficient to establish species-specific effects on the gut microbiota. Moreover, most studies have been performed in animal models or in vitro systems, frequently using chemically distinct preparations. Therefore, conclusions regarding microbiota-related effects should be interpreted cautiously. Overall, the available studies indicate that *M. alba* leaves, fruits, and their polysaccharide- or polyphenol-enriched fractions are associated with alterations in microbial composition and fermentation patterns and, in some experimental models, with changes in intestinal barrier- and inflammation-related markers [121,122,124,127,128,129]. Evidence for *M. nigra* is restricted mainly to antimicrobial or antibiofilm activity of polyphenol-rich extracts rather than whole-gut microbiota studies [126].

The following sections summarize the available evidence according to the type of mulberry-derived preparation, including whole plant materials, solvent extracts, purified fractions, and processed products, thereby reflecting the potential role of raw material and processing in shaping phytochemical composition and biological activity. Although these preparations have been evaluated in diverse experimental settings—including animal models of obesity, diabetes, metabolic syndrome, constipation, and immunosuppression, as well as in vitro fermentation and cell culture systems—this organization facilitates comparison of how different preparation strategies influence gut microbiota composition, intestinal function, and related metabolic outcomes.

### 5.1. Mulberry Leaves: Whole Matrix, Extracts, and Purified Fractions

#### 5.1.1. Whole Leaf Powder and Leaf-Based Mixtures

As discussed above, mulberry leaves constitute a complex matrix comprising both nutrients and bioactive compounds, especially alkaloids such as DNJ and antioxidant compounds [52,53,66,75]. In gut-related studies, they have been administered as whole leaf powder, formulated mixtures, water or alcohol extracts, and purified polysaccharide fractions. The biological response appears to depend strongly on whether the preparation preserves the whole matrix or enriches selected constituents.

Long-term administration of a water suspension of *Morus australis* leaf powder in healthy mice has shown that the leaf matrix can modify fecal metabolite profiles without necessarily causing large shifts in microbiota composition. In this model, the treatment altered carbohydrate and amino acid metabolism, including increased fecal maltose and decreased glucose levels, together with broad changes in amino acid-related metabolites. However, microbial diversity and SCFA production were not markedly affected. These findings suggest that, under non-diseased conditions, the whole leaf matrix may influence microbial metabolism more strongly than overall taxonomic composition [130].

It should be noted that this study was conducted using *M. australis* rather than *M. alba*, which is the species most frequently investigated in microbiota-oriented studies. Species-specific differences in phytochemical composition may therefore influence biological responses. For example, *M. australis* leaves contain relatively high amounts of fisetin, a flavonol that has been reported to modulate gut microbiota composition in experimental models of colitis and neurodegenerative disorders [131,132,133]. Nevertheless, despite the presence of this bioactive compound, administration of whole *M. australis* leaf powder to healthy mice did not induce major alterations in microbial community structure. This observation supports the view that the effects of mulberry-derived compounds on the gut microbiota may depend not only on phytochemical composition but also on the baseline physiological status of the host, with more pronounced responses often observed under dysbiotic or disease-associated conditions.

This concept is supported by findings from diabetic animal models. In streptozotocin-induced diabetic rats, dietary supplementation with mulberry leaf powder significantly improved glycaemic control, lipid metabolism, and insulin sensitivity. Treatment reduced fasting blood glucose and HbA1c levels, ameliorated dyslipidaemia and attenuated insulin resistance. These metabolic improvements were accompanied by marked modulation of gut microbiota composition. Mulberry leaf supplementation increased the abundance of Bacteroidota-associated (formerly Bacteroidetes) taxa such as *Prevotella*, *Parabacteroides*, and *Bacteroides*, together with several potentially beneficial Firmicutes members, including *Clostridium*, *Ruminococcus*, *Oscillospira*, and Ruminococcaceae. After nine weeks of treatment, the overall microbial community structure became more similar to that observed in healthy control animals, suggesting partial restoration of gut microbial homeostasis. Mechanistically, these effects were associated with attenuation of non-esterified fatty acid (NEFA) signaling and improved cellular energy metabolism [134].

Collectively, these studies suggest that whole mulberry leaf preparations exert relatively modest effects on gut microbiota under physiological conditions but may partially restore microbial homeostasis in the presence of metabolic dysfunction. However, the available evidence remains limited by the small number of studies, heterogeneity in mulberry species (including *M. australis*), disease models, and outcome measures, making it difficult to distinguish microbiota-dependent effects from broader metabolic actions of the whole leaf matrix.

#### 5.1.2. Aqueous Leaf Extracts

Aqueous extracts of *M. alba* leaves are among the best-studied mulberry preparations in relation to intestinal function. Water extraction favors polar constituents, including soluble polysaccharides, some phenolic compounds, alkaloids such as DNJ, and other hydrophilic bioactives. In rodent models of type 2 diabetes, obesity, high-fat feeding, high-fat/high-sucrose feeding, and non-alcoholic fatty liver disease, aqueous mulberry leaf extracts have repeatedly improved glycemic control, insulin resistance, lipid metabolism, hepatic inflammation, and systemic inflammatory indices.

Their gut-related effects are multifactorial: in type 2 diabetic mice, mulberry leaf water extract improved glycemia and lipid profiles while restoring dysbiotic microbial patterns [120,121]. Reported effects include normalization of Actinomycetota (formerly Actinobacteria) and Bacteroidota levels, correction of the Bacillota/Bacteroidota ratio, and modulation of genera such as *Alloprevotella*, *Parabacteroides*, and *Romboutsia*, as well as members of the family Muribaculaceae and the order Gastranaerophilales [120]. These taxonomic changes were accompanied by shifts in fecal metabolites, including branched-chain amino acid (BCAA)-related metabolites, suggesting coordinated regulation of microbiota and host metabolism. The effects of mulberry leaf water extract on gut microbiota were recently investigated in a mouse model of type 2 diabetes (streptozotocin-induced) in a study that simultaneously evaluated a 50% hydroethanolic extract prepared from the same plant material [121]. Phytochemical characterization demonstrated higher total flavonoid and chlorogenic acid contents in the hydroethanolic extract. Both preparations were associated with increased fecal SCFA concentrations, enrichment of beneficial taxa such as *Akkermansia*, *Bifidobacterium*, *Dubosiella*, and Muribaculaceae, reduced abundance of bacteria associated with lipopolysaccharide production, or metabolic inflammation. These effects were accompanied by enhanced expression of intestinal tight junction proteins (ZO-1 and occludin), reduced colonic inflammatory signaling through the TLR9–MyD88–IFN-γ pathway, activation of colonic GPR43 and GPR109A, and stimulation of hepatic AMPK. This pattern links microbial fermentation products with intestinal receptor signaling and systemic metabolic improvement. However, the published analyses reported statistical comparisons only relative to the control groups and did not directly compare the aqueous and hydroethanolic extracts. Therefore, it remains unclear whether the observed differences in phytochemical composition translated into meaningful differences in gut microbiota modulation or metabolic effects. Additional evidence indicates that modulation of gut microbiota by MLWE may also influence BCAA homeostasis. In diabetic mice, MLWE reduced serum and fecal BCAA concentrations, modulated bacterial genera associated with BCAA metabolism, suppressed the predicted microbial BCAA biosynthetic capacity and enhanced tissue-specific expression of host BCAA-catabolizing enzymes. The persistence of beneficial effects on BCAA catabolism in germ-free-mimic diabetic mice further suggests that regulation of BCAA homeostasis involves both microbiota-dependent and host-mediated mechanisms [135].

A further mechanistic axis involves endotoxemia and endocannabinoid signaling [136]. In diabetic mice, mulberry leaf water extract reduced serum lipopolysaccharide and altered endocannabinoid components such as anandamide and 2-arachidonoylglycerol. It also modulated bacterial genera associated with lipopolysaccharide and endocannabinoid metabolism, including *Acetatifactor*, *Anaerovorax*, *Bilophila*, *Colidextribacter*, *Dubosiella*, *Oscillibacter*, and *Rikenella*. Microbiota-dependency was supported in this study using germ-free mimic models, indicating that the metabolic effects of the extract were not purely host-directed but at least partly mediated through the gut microbial ecosystem. In high-fat diet and obesity-related models, hot-water extract of processed mulberry leaf tea reduced weight gain, dyslipidemia, hepatic steatosis, intestinal inflammation, and systemic inflammatory markers [137]. These effects were associated with normalization of the Bacillota/Bacteroidota ratio, decreased plasma lipopolysaccharide, altered obesity-associated genera such as *Alistipes*, enrichment of beneficial bacteria including *Alloprevotella*, Muribaculaceae, *Bifidobacterium*, and *Lactobacillus*, increased fecal SCFAs, and improved markers of barrier function such as diamine oxidase, endotoxin concentration, tight junction proteins, and mucus-related parameters. Comparable findings were reported for another aqueous *M. alba* leaf extract evaluated in HFD-fed mice [125]. Consistent with previous studies, the extract improved obesity-related metabolic disturbances, promoted enrichment of beneficial taxa including *Bifidobacterium* and was associated with SCFA remodeling, particularly increased propionate levels, further supporting modulation of the gut microbiota–SCFA axis as a common mechanism underlying the metabolic benefits of aqueous mulberry leaf extracts. Notably, this study also included a methanolic extract prepared from mulberry twigs. Although the high-dose twig extract induced more pronounced microbiota remodeling, including reductions in Bacillota and *Enterococcus* together with enrichment of *Faecalibaculum* and *Bifidobacterium*, these findings should not be interpreted as evidence for superior efficacy of methanolic extraction. Because both the extraction solvent and the plant material differed (methanolic twig extract versus aqueous leaf extract), the observed differences may reflect intrinsic phytochemical differences between twigs and leaves as well as extraction-dependent variation in composition.

Taken together, aqueous leaf extracts appear to act through a broad gut–liver–metabolic axis: restoration of microbial balance, increased production or signaling of SCFAs, strengthening of tight junction and mucus barrier function, lowering of endotoxemia, and downregulation of inflammatory pathways.

#### 5.1.3. Ethanolic and Methanolic Extracts

Ethanolic and methanolic extracts differ from aqueous extracts in their enrichment of less polar phenolic compounds, flavonoids, and other secondary metabolites. Ethanolic *M. alba* leaf extracts have been characterized by HPLC and LC-MS and tested in diabetic rodent models. In streptozotocin-induced diabetic rats, ethanol leaf extract partially normalized dysbiotic microbiota. At the phylum level, it reduced pathological Actinobacteriota overgrowth and modestly restored Bacteroidota, although Bacillota increased. At the genus level, it reversed diabetes-induced elevations in *Bifidobacterium*, *Ruminococcus 2*, *Romboutsia*, and *Lactobacillus* toward healthier profiles. Metagenomic analyses demonstrated a clear shift away from the diabetic microbial community structure, although complete normalization was not achieved. Interestingly, these microbiota changes were accompanied by restoration of liver glycogen architecture, which became structurally fragile in diabetic animals. The authors suggested that the metabolic benefits of the extract may be linked not only to improved glycaemic control but also to alterations in the gut microbial ecosystem [138]. A complementary mechanism has been proposed in db/db mice, where mulberry leaf ethanol extract was shown to modulate bacterial taxa involved in bile acid metabolism, increase fecal ω-muricholic acid concentrations, inhibit intestinal farnesoid X receptor (FXR) signaling and enhance circulating glucagon-like peptide-1 (GLP-1) levels. The persistence of FXR inhibition and GLP-1 elevation in antibiotic-treated mice further suggested that these metabolic effects may involve both gut microbiota-dependent and host-mediated mechanisms [139].

Evidence from two related studies suggests that combined mulberry leaf fractions are more effective against obesity-related outcomes than alcohol-derived polyphenol extract alone, and that this effect is linked to shifts in gut microbiota and host lipid metabolism. In the studies by Li et al. [140] and Liao et al. [129], a preparation containing mulberry leaf polyphenols and fiber (1:4, *w*/*w*) consistently showed the greatest weight-loss efficacy among the tested preparations, supporting synergistic interactions between the phenolic and fiber fractions. Notably, its effects also exceeded those of whole leaf powder. Mechanistically, the combined preparation produced more pronounced modulation of the gut microbiota, including preservation of microbial diversity, reduction in the Bacillota/Bacteroidota ratio and Lachnospiraceae abundance, and enrichment of beneficial taxa such as *Lactobacillus johnsonii* [129]. In parallel, metabolomic analyses demonstrated partial normalization of amino acid and oligopeptide metabolism, supporting coordinated regulation of microbial and host metabolic pathways [140]. The authors proposed that recombining the fiber fraction with the phenolic-rich fraction enhanced the bioaccessibility and bioavailability of both components, thereby contributing to the superior anti-obesity efficacy of the combined preparation compared with either whole leaf powder or the isolated fractions. Collectively, these findings support the concept that selective enrichment of complementary functional constituents, while preserving their functional interactions, may provide greater biological activity than either isolated fractions or the native leaf matrix.

#### 5.1.4. Leaf Polysaccharides and Oligosaccharides

Among available preclinical studies, purified mulberry leaf polysaccharides provide some of the clearest evidence for prebiotic-like effects. Water-soluble leaf polysaccharides administered to obese mice reduced adiposity, improved insulin resistance, alleviated colonic lesions, remodeled gut microbial communities and altered plasma lipid profiles [122]. Specific correlations between bacterial taxa and lipid metabolites suggest that polysaccharide-driven microbiota modulation contributes to improved host lipid metabolism.

In immunosuppressed mice, crude mulberry leaf polysaccharide restored thymus and spleen indices [141], improved intestinal barrier damage, regulated cytokine levels and restored SCFAs, including acetate, propionate, and butyrate. At the microbiota level, it increased increased Bacteroidota and decreased Bacillota, *Butyricimonas*, and *Eubacterium*. This is notable because it links polysaccharide intake not only with metabolic regulation but also with immune recovery and barrier repair.

Defined leaf polysaccharide fractions have been used to examine structure–function relationships. The SY01-21, SY01-22, and SY01-23 fractions differ in molecular weight and monosaccharide composition. In in vitro *Bacteroides* culture systems, SY01-21 selectively supported *Bacteroides cellulosilyticus* and SY01-22 supported *Bacteroides ovatus*, while the low-molecular-weight fraction SY01-23 supported both species and promoted acetate and propionate production. These findings show that polysaccharide molecular size and monosaccharide composition determine which bacteria can utilize the substrate and which SCFAs are produced [142].

Simulated gastrointestinal digestion further modifies polysaccharide function. A defined mulberry leaf polysaccharide fraction, MLP-2, and its intestinally digested form, MLP-2I, have been tested in human fecal fermentation models. Digestive processing altered molecular weight and composition, enhanced α-amylase and α-glucosidase inhibitory activity, lowered pH during fermentation and increased acetate, propionate, and butyrate production. This suggests that the biological activity of mulberry polysaccharides should not be inferred solely from the native extract composition, because the digested colonic substrate may be structurally and functionally different [143].

Mulberry leaf oligosaccharides also show selective fermentation. A defined oligosaccharide improved metabolic phenotypes in obese or diabetic mice was fermented to lactate, acetate, and butyrate, and promoted taxa such as *Ligilactobacillus* and *Lactobacillus murinus*. These results support the broader concept that lower-molecular-weight carbohydrates from mulberry leaves may function as selective microbial substrates [24].

Enzymatic hydrolysis of mulberry leaf polysaccharides may generate oligosaccharides with enhanced prebiotic properties and the ability to modulate gut microbiota. In two studies, Hu et al. isolated non-digestible oligosaccharide fractions (MLO 1-2 and MLO 2-1) that resisted simulated gastrointestinal digestion, suggesting their potential to reach the colon intact [24,144]. In pure-culture experiments, MLO 1-2 promoted the growth of probiotic strains, including *Bifidobacterium bifidum*, *B. adolescentis*, *Lacticaseibacillus rhamnosus*, and *Lactobacillus acidophilus*, while increasing acetate and lactate production. In contrast, MLO 2-1 was evaluated using an in vitro fecal fermentation model, where it increased acetate and butyrate production and selectively enriched *Ligilactobacillus murinus*, a commensal bacterium reported to be depleted in diabetic mice. Furthermore, both MLO 2-1 and *L. murinus* exerted hypoglycaemic effects in animal experiments. Collectively, these findings indicate that enzymatic processing of mulberry polysaccharides can generate microbiota-accessible oligosaccharides with prebiotic activity and potential metabolic benefits mediated through gut microbial modulation.

Overall, the available literature supports a fairly consistent conclusion that mulberry leaf polysaccharides act as microbiota-accessible carbohydrates that increase SCFA production, modulate specific bacterial taxa and improve host metabolic, barrier, and immune phenotypes in preclinical models. However, the magnitude and nature of these effects appear to depend on the structural characteristics of the isolated carbohydrates. Digestion and enzymatic processing modify molecular weight and fermentation properties, thereby influencing microbial substrate utilization and the resulting SCFA profile.

Across preparation types—from whole leaf matrix to solvent extracts and defined fractions—the key microbiota and gut-related outcomes are summarized in Table 1.

### 5.2. Mulberry Fruits: Whole Fruits, Polysaccharides, Polyphenols, and Combined Fractions

#### 5.2.1. Whole Fruit Matrix

The whole fruit matrix differs fundamentally from leaves, because it contains a higher proportion of readily available carbohydrates and fruit-specific phenolics, particularly anthocyanins in dark-colored fruits. Gut-related studies have examined whole fruit, purified fruit polysaccharides, polyphenol-rich fractions, combined polyphenol–polysaccharide fractions, fermented fruit, and fruit pomace.

Whole mulberry fruit has been investigated in constipation models, particularly using *M. atropurpurea* [145]. Oral administration increased fecal water content, accelerated intestinal transit, improved colon mucus cell counts, reduced inhibitory neurotransmitters such as nitric oxide and vasoactive intestinal peptide, and increased excitatory mediators including acetylcholine, substance P, and motilin. It also increased several SCFAs, including acetate, propionate, butyrate, valerate, and isovalerate. Microbiologically, whole fruit increased fecal *Lactobacillus* and *Bifidobacterium* and decreased *Helicobacter* and Prevotellaceae. These findings indicate that whole mulberry fruit can affect motility, mucus barrier function, microbial fermentation, and neurochemical regulation of intestinal transit.

Beyond whole fruit preparations, a functional mulberry juice enriched with isomaltooligosaccharides (IMO) has also been evaluated in an in vitro fecal fermentation model. The non-thermally pasteurized juice promoted microbial fermentation in an IMO dose-dependent manner, resulting in substantial increases in propionate and butyrate production after 48 h of incubation. The relative abundance of bifidobacteria increased significantly, reaching 5.03% and 17.53% after 24 h fermentation in juices supplemented with 2% and 8% IMO, respectively. Fermentation also generated several anthocyanin-derived metabolites, including 3-(2-hydroxyphenyl)propionic acid and 3,4-dihydroxybenzaldehyde, indicating active microbial biotransformation of juice polyphenols. These findings suggest that supplementation of mulberry juice with IMO may enhance its prebiotic potential by promoting beneficial gut bacteria and increasing the production of health-related microbial metabolites [146].

#### 5.2.2. Fruit Polysaccharides

Fruit polysaccharides have been investigated in both animal and in vitro human fecal fermentation systems. In human fecal fermentation, *M. alba* fruit polysaccharides composed of arabinose, galactose, glucose, rhamnose, and galacturonic acid were substantially utilized by the microbiota. Approximately 45% of carbohydrate was consumed over 48 h, pH decreased, and total SCFAs increased markedly, including acetate, propionate, and butyrate. The microbial community shifted toward increased Bacteroidota and decreased Bacillota. Specific monosaccharides were associated with individual fermentation products; for example, galactose and galacturonic acid were linked with acetate and butyrate, whereas arabinose and glucose were linked with propionate production. These observations reinforce the importance of monosaccharide composition in shaping fermentation profiles [128]. In diabetic db/db mice, purified black mulberry fruit polysaccharide obtained by hot-water extraction followed by deproteinization, decolorization, ethanol precipitation, and dialysis decreased weight gain and hyperglycemia. The resulting heteropolysaccharide contained arabinose, galactose, glucose, rhamnose, and galacturonic acid and consisted of several molecular-weight fractions ranging from approximately 21 to 210 kDa. Treatment enriched beneficial genera such as Bacteroidales, *Lactobacillus*, *Allobaculum*, *Bacteroides*, and *Akkermansia*, while shifting the Bacteroidota/Bacillota balance toward healthier values. A related approach was reported by Li et al. [147], who investigated a purified mulberry galacto-oligosaccharide (MGO) obtained from enzymatically hydrolyzed polysaccharides isolated from *M. nigra* fruits. The resulting oligosaccharide consisted predominantly of galactose and had an average molecular weight of approximately 987 Da. In a mouse model of T2DM, MGO supplementation improved hyperglycemia, insulin resistance, and oxidative stress, while simultaneously modulating gut microbiota composition. Specifically, MGO increased the abundance of Prevotellaceae and *Lactobacillus* and reduced Lachnospiraceae, changes that were accompanied by activation of the hepatic PI3K/Akt signaling pathway and improved glucose metabolism. These findings suggest that oligosaccharides derived from mulberry fruit polysaccharides may exert both prebiotic and metabolic effects, potentially complementing the microbiota-modulating activity observed for higher molecular weight fruit polysaccharides.

In high-fat diet-induced metabolic syndrome, *M. alba* fruit polysaccharide fractions improved metabolic markers and colon histology, enriched beneficial taxa such as *Muribaculum* and Lachnospiraceae NK4A136 group, and reduced genera including *Prevotella* 2, *Bacteroides*, *Faecalibacterium*, and *Fusobacterium* [124]. The tested fraction was obtained by hot-water extraction followed by deproteinization and ethanol precipitation, yielding a polysaccharide-enriched preparation. Such processing steps may contribute to the enrichment of fermentable polysaccharides while reducing non-polysaccharide components, thereby influencing the observed microbiota responses. Support for this interpretation comes from an in vitro human fecal fermentation study using polysaccharides isolated from black mulberry fruits by different extraction approaches, including hot-water extraction and enzyme-assisted extraction with pectinase, pectin lyase, cellulase, or mixed enzymes. The resulting polysaccharide fractions differed in structural characteristics and fermentation behavior. Although all preparations were utilized by the gut microbiota and increased SCFA production to varying degrees, water-extracted and pectin lyase-extracted polysaccharides showed the highest fermentability and prebiotic potential. These fractions promoted the growth of Bacteroidota and Bacillota while suppressing Pseudomonadota (formerly Proteobacteria) and Fusobacteriota, indicating that extraction-induced structural differences can influence microbial accessibility and subsequent microbiota responses [148]. Collectively, these findings suggest that the microbiota-modulating properties of mulberry fruit polysaccharides depend not only on their monosaccharide composition and molecular characteristics but also on the extraction technology used to obtain them. Apparent inconsistencies among studies should be interpreted with caution because microbiota analyses are often reported at different taxonomic resolutions. For example, increases in Bacteroidales reported in diabetic db/db mice and reductions in *Bacteroides* observed in high-fat diet models do not necessarily represent contradictory findings, as *Bacteroides* constitutes only one genus within the broader Bacteroidales order [124,147]. Moreover, differences in extraction procedures, polysaccharide structure, host phenotype, and experimental design may further contribute to variation in microbiota responses. Therefore, some reported discrepancies are likely methodological or context-dependent rather than indicative of fundamentally different biological effects.

#### 5.2.3. Fruit Polyphenols and Combined Polyphenol–Polysaccharide Fractions

Fruit polyphenol fractions and combined polyphenol–polysaccharide preparations have demonstrated microbiota-related and metabolic benefits in several experimental models. Importantly, the available studies indicate that these effects may depend not only on the presence of polyphenols themselves but also on the way fruit constituents are fractionated and processed.

In high-fat diet-fed mice a combined polyphenol–polysaccharide fraction improved features of metabolic syndrome and proximal colon histology [124]. However, the combined fraction produced the most robust effects, more strongly enriching beneficial taxa such as *Muribaculum* and the Lachnospiraceae NK4A136 group, reducing potentially unfavorable genera, and generating a distinct fecal metabolite signature involving 23 metabolites. The causal contribution of the microbiota was further supported by fecal microbiota transplantation experiments. Transfer of microbiota from mice treated with the combined mulberry fruit polyphenol–polysaccharide fraction into pseudo-germ-free high-fat diet-fed recipient mice improved dyslipidemia, organ injury, and proximal colon injury and activated PPARα/PGC-1α signaling, indicating that at least part of the biological activity of the fraction was microbiota-mediated [123].

The superiority of combined preparations is biologically plausible because polysaccharides provide fermentable substrates for microbial growth and SCFA production, whereas polyphenols can selectively influence microbial populations, undergo microbial biotransformation into bioactive metabolites and modulate inflammatory and oxidative pathways. Their combination may therefore influence both microbial ecology and host signaling more effectively than either fraction alone [123,124].

Distinct microbiota-related effects have also been reported for purified fruit polyphenol preparations. In db/db mice, a purified *M. alba* fruit polyphenol extract improved glucose homeostasis, pancreatic histology, and antioxidant status while simultaneously remodeling both small and large intestinal microbiota [149]. Interestingly, the microbial response differed between intestinal regions. Changes in the small intestinal microbiota were more closely associated with blood glucose and insulin regulation, whereas alterations in the large intestinal microbiota were linked primarily to carbohydrate metabolism and SCFA production. Polyphenol treatment decreased Bacillota, *Lactobacillus*, and Bacilli while increasing Bacteroidota, accompanied by elevated propionate and butyrate concentrations. These findings suggest that purified mulberry polyphenols may exert region-specific microbiota-mediated metabolic effects and highlight the importance of considering intestinal compartments beyond fecal samples alone.

The influence of processing strategy is further illustrated by a study using a polyphenol-rich extract from *M. nigra* fruits obtained through supercritical carbon dioxide extraction [107]. Unlike conventional solvent extracts, supercritical CO_2_ extraction may preferentially enrich less polar phenolic constituents and thus generate a phytochemical profile that differs from that of the native fruit matrix. In a rat model of Alzheimer’s disease induced by D-galactose and aluminum chloride, this extract improved cognitive performance, reduced hippocampal Aβ accumulation and attenuated neuroinflammation. These effects were accompanied by restoration of microbial diversity, enrichment of SCFA-producing Bacillota taxa, and modulation of bile acid and lipid metabolism. Untargeted metabolomic analysis identified β-muricholic acid, docosahexaenoic acid (DHA), and ergosterol as key metabolites associated with the response, supporting a role for gut microbiota–host co-metabolism in the observed neuroprotective effects.

Collectively, these findings indicate that the microbiota-modulating properties of mulberry fruit polyphenols depend not only on the polyphenols themselves but also on the composition of accompanying fractions and the extraction technology used to obtain them. Combined polyphenol–polysaccharide preparations, purified polyphenol extracts, and supercritical CO_2_-derived fractions differ substantially in composition and may therefore influence gut microbial communities and host metabolism through distinct mechanisms.

Overall, the key microbiota and intestinal outcomes associated with mulberry fruit polysaccharides are summarized in Table 2.

#### 5.2.4. Functional Mulberry Juice and Fruit Pomace

Functional mulberry juice combined with isomaltooligosaccharides has been studied in human fecal fermentation models. Increasing isomaltooligosaccharide content enhanced propionate and butyrate production, markedly increased *Bifidobacterium*, and generated phenolic and anthocyanin metabolites. This model illustrates how fruit-derived polyphenols and added fermentable oligosaccharides may act together to produce a symbiotic-like fermentation pattern. Mulberry pomace, a by-product of juice or fruit processing, contains residual fiber and polyphenols and has been investigated particularly after fermentation. *Lactobacillus*-fermented mulberry pomace increased antioxidant capacity and promoted beneficial bacteria including *Lactobacillus*, *Bifidobacterium*, *Ruminococcus*, *Akkermansia*, and butyrate-producing taxa, while reducing *Escherichia coli* and *Enterococcus*. It also increased total SCFAs and lactic acid. These results suggest that mulberry pomace may be a valuable functional by-product, especially when fermentation improves the release or transformation of bioactive compounds [22].

### 5.3. Fermentation as a Modifier of Mulberry Bioactivity

#### 5.3.1. Fermented Leaves

Fermentation substantially modifies the mulberry matrix [131,132,133,134]. Depending on the microorganisms used, fermentation may reduce antinutritional factors such as tannins, increase protein availability, transform phenolic compounds, enrich organic acids and improve antioxidant activity. It may also markedly increase the concentration of γ-aminobutyric acid (GABA), with approximately 6.7- to 10.5-fold increases reported in fermented white mulberry leaf preparations [73,74]. These compositional changes can alter digestibility, microbial fermentation, and intestinal responses [22,151].

Fermentation may further enhance the gut-related effects of mulberry leaves by increasing the bioaccessibility of phenolic compounds and modifying their interactions with gut microbiota. In an in vitro digestion and colonic fermentation study, solid-state fermented mulberry leaves exhibited higher phenolic bioaccessibility, antioxidant activity, and SCFA production than unfermented leaves [152]. Fermented samples increased acetate production and reduced the Bacillota-to-Bacteroidota ratio, whereas unfermented mulberry leaves preferentially promoted the growth of *Bifidobacterium*. These findings suggest that fermentation can alter both the availability of phenolic constituents and the microbiota-modulating properties of mulberry leaves, potentially enhancing their capacity to support gut health.

Fermentation-based processing may also influence the gut-related effects of mulberry by enhancing the recovery of specific bioactive compounds. In a study using mixed fermentation with *Lactobacillus fermentum* and *Saccharomyces cerevisiae*, the extraction yield of 1-deoxynojirimycin (DNJ) increased from 3.24 to 5.97 mg/g. Administration of purified DNJ to diabetic mice improved glucose homeostasis and insulin sensitivity while partially restoring gut microbial balance. DNJ supplementation increased the abundance of *Lactobacillus*, *Bifidobacterium*, *Oscillibacter*, *Alistipes*, and members of the Lachnospiraceae family, while reducing taxa enriched in diabetic animals, including *Klebsiella*, *Ruminococcus*, *Weissella*, and selected Prevotellaceae groups [153]. Unlike studies using crude leaf powders or conventional extracts, this work demonstrates that biotechnological processing can substantially increase the recovery of a specific bioactive constituent, thereby indirectly influencing microbiota-related outcomes through changes in phytochemical composition.

Fermented mulberry leaves have been studied in pigs, sows, and fish. In pigs, fermented mulberry leaves increased beneficial taxa such as Actinobacteria, *Bifidobacterium*, and *Prevotella*, while reducing Proteobacteria and *Streptococcus*. They also decreased fecal odor compounds including ammonia, indole, and skatole, suggesting reduced proteolytic fermentation and improved colonic metabolic activity. In gestating sows, fermented leaves increased microbial diversity, raised Bacteroidota, lowered Bacillota and altered SCFA metabolism. Fermented mulberry leaves also improve intestinal structure and immune markers. In pig studies, they increased villus height and the villus height-to-crypt depth ratio, improved digestibility, enhanced ileal antioxidant capacity, increased mucin and tight junction-related markers, and downregulated inflammatory pathways such as TLR4/NF-κB, TNF-α, and MyD88-dependent signaling. These changes indicate that fermentation-enhanced leaf matrices may improve both microbial ecology and epithelial barrier function. In crucian carp, fermented mulberry leaves incorporated into feed at 5% improved growth, serum antioxidant status, and intestinal digestive enzyme activities with minimal disruption of microbiota. However, inclusion levels of 10% or higher impaired growth and disturbed microbial homeostasis. This dose-dependent response is important because it shows that fermented products are not universally beneficial at all concentrations [154]. The matrix, dose, animal species, and background diet determine whether fermentation improves or disrupts intestinal ecology.

Fermented mulberry leaf beverages have also been investigated, although evidence for direct microbiota effects remains limited. Mulberry (*M. alba*) leaf tea kombucha produced by a symbiotic consortium of acetic acid bacteria and yeasts exhibited antioxidant, anti-inflammatory, antihyperglycemic, and antihypertensive activities in digestion, cell, and enzyme assays. However, microbiota-related endpoints and in vivo confirmation are currently lacking [155].

#### 5.3.2. Fermented Fruits and Fruit Products

Fermented mulberry fruit preparations have been developed to reduce simple sugars while retaining anthocyanins and dietary fiber. Fermentation substantially alters the chemical composition of mulberry fruit products and may thereby modify their interactions with gut microbiota. One important consequence is the reduction in readily available sugars while preserving or enhancing the concentration of other bioactive compounds. For example, fermentation of *Morus atropurpurea* fruits was successfully used to remove fructose and glucose while retaining hypoglycemic activity. In diabetic mice, the resulting preparation improved glucose homeostasis, insulin-related parameters, dyslipidemia, oxidative stress, and organ injury. These effects were accompanied by increased SCFA production and enrichment of beneficial genera including *Lactobacillus, Bifidobacterium*, and *Akkermansia*, suggesting that fermentation can improve the suitability of fruit-derived products for metabolic disorders by reducing sugar content while maintaining microbiota-related functionality [156].

Fermentation may also enhance the release, transformation, and microbial accessibility of phenolic compounds. Fermentation of mulberry pomace with *Lactobacillus plantarum* increased antioxidant activity, promoted the release and biotransformation of phenolics, enhanced SCFA production and shifted the microbial community toward a more favorable composition characterized by increased abundance of *Lactobacillus*, *Bifidobacterium*, *Ruminococcus*, and *Akkermansia* [22]. Similarly, fermentation of mulberry juice with *Lactobacillus plantarum* SC-5 increased phenolic acid metabolites and was associated with enrichment of Bifidobacteriaceae and Lactobacillaceae, enhanced SCFA production and improved cognitive function in an ageing mouse model [157]. These findings suggest that microbial biotransformation of fruit polyphenols may contribute substantially to the biological activity of fermented mulberry products.

Fermentation may also substantially increase the concentration of γ-aminobutyric acid (GABA), another bioactive constituent of mulberry fruits. Fermented mulberry juices have been reported to contain 3.31–7.48 g/L GABA depending on the microbial strains and fermentation conditions employed, highlighting the strong impact of microbial processing on the phytochemical and functional profile of mulberry-derived products [158,159].

Mulberry ferment preparations have also been investigated in constipation models. During fermentation, total polyphenol and flavonoid contents increased together with antioxidant activity. In constipated mice, administration of mulberry ferment shortened the first black fecal defecation time, accelerated gastrointestinal transit, increased fecal moisture content, alleviated ileal injury and modified gut microbiota composition by increasing Bacteroidales and Bacteroidales_S24-7 while reducing *Oscillibacter* and *Desulfovibrio* [145]. These findings suggest that fermentation-induced compositional changes may contribute to improved intestinal motility and barrier function through modulation of microbial metabolism and suppression of potentially pro-inflammatory or sulfide-producing taxa.

Fermented mulberry beverages have mainly been assessed in vitro. Mulberry vinegar suppressed LPS/IFN-γ-induced nitric oxide, reactive oxygen species and inflammatory cytokines through NF-κB inhibition in glial cells, but these data cannot yet be translated into gut microbiota effects [160]. Similarly, *Bacillus*-fermented mulberry-derived postbiotics show antioxidant, anti-inflammatory, and antibacterial activity in vitro, but have not yet been tested in human gut microbiota or clinical models [161].

#### 5.3.3. Fermented Versus Unfermented Preparations

Across available animal and in vitro models, both fermented and unfermented mulberry preparations can support beneficial microbes and intestinal function. However, fermentation tends to enhance bioactive release, reduce antinutritional factors, increase organic acid and SCFA-related outcomes and promote probiotic-like taxa. Fermented products more consistently improve villus structure, tight junction, mucin-related markers, immune indices, and microbial profiles associated with SCFA production. Nevertheless, fermentation does not automatically guarantee benefit: high inclusion levels of fermented leaves in fish disturbed microbiota and impaired performance. Therefore, fermented mulberry preparations require dose optimization and matrix-specific validation.

## 6. Intestinal Barrier, Inflammation, and Functional Outcomes

### 6.1. Tight Junctions, Mucus, and Endotoxemia

A recurrent finding across mulberry studies is the improvement of multiple barrier-related endpoints, including tight junction- and mucus-associated markers, intestinal morphology, mucosal immunity, and inflammatory responses. A particularly comprehensive demonstration of the interplay between gut microbiota, microbial metabolism, and intestinal barrier integrity was provided by Song et al. [127]. In weaned piglets, dietary supplementation with white mulberry leaf extract (0.1% of the diet) increased villus height and villus-to-crypt ratio, upregulated the expression of ZO-1, claudin-1, and MUC2, reduced circulating lipopolysaccharide levels and attenuated local inflammatory responses. These changes were accompanied by increased abundance of *Bifidobacterium* and *Lactobacillus*, reduced numbers of *Escherichia coli*, enhanced production of lactate, acetate, butyrate, and total SCFAs, and decreased concentrations of potentially harmful protein fermentation metabolites, including p-cresol, skatole, histamine, and tryptamine. The findings suggest that barrier strengthening may be closely linked to favorable shifts in microbial composition and metabolism.

Similar effects were observed following supplementation with fermented mulberry leaves (10% of the diet) [151]. Fermentation, applied to reduce antinutritional factors and improve the bioavailability of bioactive constituents, increased villus height and villus-height-to-crypt-depth ratio while reducing crypt depth throughout the small intestine of finishing pigs. Fermented mulberry leaves also enhanced mucosal antioxidant capacity, upregulated MUC1 and claudin-2 expression, improved mucosal immunoglobulin levels and downregulated key inflammatory signaling mediators, including TLR4, NF-κB, and MyD88. These findings indicate that microbial fermentation may further strengthen the gut-supporting properties of mulberry leaves through combined effects on intestinal morphology, mucosal immunity, and inflammatory signaling.

The influence of formulation strategies on gut-related bioactivity was further explored by Kim et al. [162], who developed a hot-melt extruded sustained-release formulation of *M. alba* leaves containing rutin and isoquercitrin in order to improve the solubility and bioavailability of these poorly water-soluble flavonoids. The resulting preparation inhibited the growth of pathogenic bacteria while supporting probiotic strains and, in an LPS-challenged Caco-2/RAW264.7 co-culture model, restored transepithelial electrical resistance, increased tight junction protein expression and reduced pro-inflammatory cytokine production. These results indicate that technological processing may modify the bioactivity of mulberry preparations and contribute to improved barrier-supporting properties.

Additional evidence from animal studies suggests that mulberry-derived polysaccharides may contribute to the maintenance of intestinal barrier integrity. In cyclophosphamide-treated chicks, mulberry leaf polysaccharides attenuated jejunal injury and upregulated the expression of key barrier-related proteins, including Claudin-1, ZO-1, and MUC2, while simultaneously restoring gut microbial homeostasis. These effects were accompanied by improved immune responses and antioxidant status, indicating a multifaceted role of mulberry polysaccharides in preserving intestinal health [163].

The barrier-protective effects were also observed for an anthocyanin-enriched fraction obtained from mulberry fruits through ethanol extraction followed by resin purification [164]. This preparation, composed predominantly of cyanidin-3-*O*-glucoside and cyanidin-3-*O*-rutinoside (approximately 75% of the extract), restored ZO-1, occludin, and claudin-3 expression in DSS-induced colitis, reduced intestinal inflammation and oxidative stress, and partially normalized gut microbiota composition by decreasing *Escherichia-Shigella* and increasing *Akkermansia*, Muribaculaceae, and *Allobaculum*. These findings suggest that anthocyanins represent one of the mulberry-derived compound classes potentially involved in the observed barrier-protective and microbiota-modulating effects.

Evidence for barrier-modulating activity has also been reported for DNJ, a characteristic mulberry alkaloid. In fattening rabbits, dietary DNJ supplementation increased the expression of several tight junction- and mucus-related genes, including occludin, claudin-1, claudin-2, JAM2, JAM3, mucin4, and mucin6, while reducing inflammatory markers and altering gut microbial composition [165]. However, the higher supplementation level was associated with reduced villus height and impaired growth performance, suggesting that some intestinal responses to DNJ may be dose-dependent.

Collectively, these findings suggest that mulberry-derived preparations may reduce intestinal permeability and metabolic endotoxemia through multiple complementary mechanisms. Beyond direct effects on tight junction proteins and mucus-associated pathways, mulberry preparations may influence intestinal morphology, mucosal immunity, microbial metabolism, and inflammatory signaling. Improvements in barrier-related markers are frequently accompanied by reductions in circulating lipopolysaccharide and pro-inflammatory mediators, supporting the hypothesis that microbiota remodeling and barrier strengthening act together.

Importantly, the magnitude and nature of these effects appear to depend on the type of mulberry preparation, processing strategy and dosage, with fermentation, extraction, purification, and formulation technologies influencing the composition and bioactivity of the resulting products.

### 6.2. SCFAs as Mediators

Short-chain fatty acids are central mediators connecting mulberry polysaccharides, oligosaccharides, fruit fibers, fermented products, and host physiology [127,128,143]. Acetate, propionate, and butyrate increased in several models, including human fecal fermentation of fruit polysaccharides, fermentation of leaf polysaccharide fractions, diabetic mice treated with leaf extracts, piglets supplemented with leaf extract, and animals receiving fermented mulberry products.

These metabolites are relevant, because they support colonocyte metabolism, strengthen tight junctions, promote mucus production, and regulate immune balance and signal through G-protein coupled receptors such as GPR43 and GPR109A [121,127]. In mulberry studies, activation of colonic GPR43/GPR109A and hepatic AMPK provides a mechanistic link between microbial fermentation and systemic metabolic improvements.

However, SCFA responses are not uniform and appear to depend strongly on the type of mulberry preparation and processing strategy employed. Han et al. [166] demonstrated that mulberry leaf water extracts, alkaloid fractions, and polysaccharide fractions differed substantially in their ability to stimulate acetate, propionate, and butyrate production in diabetic mice. Likewise, enzymatic hydrolysis of mulberry leaf polysaccharides generated oligosaccharide fractions with distinct fermentation characteristics and microbiota-modulating properties [144]. Similar observations have been reported for fruit-derived polysaccharides, where extraction procedures influenced fermentability and SCFA production during human fecal fermentation [148]. Fermentation-based processing may further enhance SCFA generation, as demonstrated for solid-state fermented mulberry leaves [152], fermented mulberry pomace [22], and fermented mulberry juice [157], all of which exhibited increased SCFA production together with favorable shifts in microbial composition.

Taken together, the available evidence indicates that SCFAs represent one of the most consistent mechanistic links between mulberry-induced changes in gut microbiota and host physiological responses. The quantity and profile of SCFAs produced depend not only on the microbial community but also on the composition and processing history of the mulberry-derived substrate, including extraction, purification, enzymatic modification, and fermentation. Representative changes in SCFA production reported for different mulberry-derived preparations are summarized in Table 3.

### 6.3. Inflammatory Signaling and Immune Modulation

The anti-inflammatory effects of mulberry preparations appear closely interconnected with the improvements in intestinal barrier integrity, microbial composition, and microbial metabolism described above. Reduced intestinal permeability may limit the translocation of microbial products such as lipopolysaccharide, thereby decreasing activation of innate immune pathways and downstream inflammatory signaling. In parallel, changes in gut microbial composition and microbial metabolite production may further contribute to the regulation of mucosal immune responses.

Evidence from experimental studies suggests that mulberry-derived preparations can influence both inflammatory signaling pathways and intestinal immune homeostasis. In piglets, supplementation with white mulberry leaf extract reduced serum TNF-α and IL-1β concentrations, increased secretory IgA levels, and improved microbial and metabolic profiles [127]. In immunosuppressed mice, mulberry leaf-derived polysaccharides restored immune organ indices, regulated cytokine production and reshaped gut microbial communities, supporting interactions between gut microbiota and mucosal immune responses [141].

Several studies have also identified specific inflammatory pathways affected by mulberry-derived preparations. Leaf extracts downregulated colonic inflammatory signaling involving TLR9, MyD88 and IFN-γ [121], whereas fermented mulberry leaves suppressed TLR4/NF-κB/MyD88 signaling and reduced the expression of TNF-α and other pro-inflammatory mediators [151]. These findings suggest that microbiota-associated changes induced by mulberry preparations may be linked to attenuation of innate immune activation and downstream inflammatory responses.

The anti-inflammatory effects of mulberry preparations are likely mediated through multiple complementary mechanisms, including antioxidant activity, modulation of gut microbiota composition, enhancement of barrier integrity, and increased production of beneficial microbial metabolites such as SCFAs. Together, these processes may contribute to improved mucosal immune homeostasis and reduced intestinal inflammation [168,169].

Notably, the anti-inflammatory effects observed for fermented mulberry leaves [151] are consistent with broader evidence from randomized controlled trials showing beneficial metabolic and inflammatory effects of botanical fermented foods [170]. However, this evidence cannot be directly extrapolated to mulberry-derived products. Although direct clinical evidence for fermented mulberry preparations is currently lacking, the available experimental data support the hypothesis that fermentation may represent a promising strategy for enhancing the gut-related bioactivity of mulberry materials.

Despite these encouraging findings, the relative contribution of microbiota remodeling, barrier protection, SCFA-mediated signaling, and direct phytochemical effects remains insufficiently understood. Future studies should aim to clarify which mulberry-derived compounds and processing strategies are primarily responsible for the observed immunomodulatory effects.

## 7. Influence of Raw Material and Processing Methods on Microbiota-Related Effects

A central theme emerging from the available evidence is that the effects of mulberry on gut microbiota and intestinal function cannot be considered independently of raw material selection and processing methods. The studies discussed throughout this review demonstrate that extraction solvent, plant part, fermentation, enzymatic hydrolysis, purification, and formulation strategies can markedly influence the composition and functionality of the resulting preparations [120,124,125,136,142,143,162].

The choice of plant material represents the first important source of variation. Whole leaves, leaf extracts, fruit-derived preparations, and purified fractions differ substantially in their content of polysaccharides, phenolics, anthocyanins, alkaloids, and dietary fiber. Moreover, species-specific differences may further contribute to variability in biological responses. For example, most microbiota-related studies have focused on *M. alba*, whereas evidence for *M. nigra* and other mulberry species remains comparatively limited.

Processing methods further modify the phytochemical composition and bioavailability of mulberry-derived products. Water extracts, typically enriched in polysaccharides, DNJ, and other hydrophilic constituents, were more frequently associated with SCFA production, barrier-supporting effects, and regulation of glucose metabolism [120,136,141,142]. In contrast, ethanol-derived preparations and purified anthocyanin fractions often exerted stronger effects on microbial composition, inflammatory responses, and oxidative stress [121,136,164]. These differences likely reflect variation in the relative abundance of polysaccharides, phenolics, and alkaloids recovered by different extraction procedures.

The importance of processing is particularly evident in studies employing fractionation and structural modification of polysaccharides. Enzymatic hydrolysis of mulberry leaf polysaccharides generated oligosaccharide fractions with distinct fermentation characteristics and selective effects on bacterial growth [142,143]. Likewise, extraction methods applied to fruit polysaccharides influenced molecular structure, fermentability, and SCFA production during human fecal fermentation, demonstrating that microbiota responses depend not only on botanical origin but also on the way polysaccharides are isolated and processed.

Fermentation represents another important strategy for modifying mulberry bioactivity. Fermented leaf and fruit preparations differed from their unfermented counterparts in phytochemical composition, phenolic bioaccessibility, GABA content, and microbiota-modulating properties [22,151,152]. Fermentation may also increase the recovery of specific bioactive compounds, such as DNJ, thereby indirectly influencing gut microbiota composition and host metabolic responses. These observations suggest that microbial biotransformation can substantially alter the functional properties of mulberry-derived products.

Technological processing may further influence gut-related activity. A hot-melt extruded formulation of mulberry leaves enriched in rutin and isoquercitrin improved barrier-related outcomes in intestinal cell models [162], while purification procedures generated anthocyanin-enriched fractions with pronounced anti-inflammatory and barrier-protective effects [164]. Such findings indicate that formulation and purification strategies can modify both the concentration and biological accessibility of active constituents.

Overall, the available evidence suggests that microbiota-related effects are determined not by a single class of compounds but by interactions among polysaccharides, phenolics, alkaloids, dietary fiber, and their microbial metabolites. In many cases, whole matrices or combined fractions exerted stronger effects than isolated constituents [123,124,129], supporting the concept that synergistic interactions within the mulberry matrix contribute substantially to biological activity. Consequently, both raw material selection and processing history should be carefully considered when comparing studies and interpreting the gut-related effects of mulberry-derived preparations.

## 8. Integrated Mechanistic Interpretation

The evidence reviewed in this work suggests that the gut-related effects of mulberry are not determined by a single bioactive compound or a single microbial target. Instead, they emerge from interactions among the phytochemical composition of the starting material, processing-induced modifications of the mulberry matrix, gut microbial metabolism, and host physiological responses.

A recurring observation across studies is that different processing strategies appear to shift the dominant mechanism through which mulberry interacts with the gut ecosystem. Preparations enriched in polysaccharides and dietary fiber primarily act as microbial substrates, promoting carbohydrate fermentation and SCFA production. In contrast, polyphenol- and anthocyanin-enriched fractions appear to exert stronger effects on microbial community composition, inflammatory pathways, and oxidative status. Alkaloid-rich preparations, particularly those containing DNJ, are more closely linked to glucose metabolism and host metabolic regulation, although their effects may also be mediated indirectly through alterations in gut microbial ecology. Conversely, gut microorganisms also actively transform mulberry phytochemicals. In vitro studies demonstrated that intestinal probiotic bacteria metabolize mulberry anthocyanins into lower-molecular-weight phenolic acids, indicating that microbial biotransformation may substantially modify the spectrum of bioactive metabolites reaching the host. Thus, the relationship between mulberry and the gut microbiota is bidirectional, with microbial metabolism influencing not only host physiology but also the chemical fate of mulberry-derived compounds [171].

The available data further indicate that processing does not simply increase or decrease biological activity but may fundamentally alter its nature. Enzymatic hydrolysis generates oligosaccharides with fermentation characteristics distinct from those of the parent polysaccharides. Fermentation modifies the accessibility of phenolics, increases concentrations of selected metabolites such as GABA or DNJ, and changes the balance between readily available and complex carbohydrates. Purification and formulation strategies can further enrich specific classes of compounds and modify their biological availability. Consequently, preparations derived from the same botanical source may act through partially different microbiota-mediated pathways.

Another important observation is that microbiota responses appear highly context-dependent. In healthy animals, whole mulberry leaf preparations often produced relatively modest alterations in microbial composition despite measurable changes in microbial metabolism. In contrast, more pronounced effects were generally observed in models characterized by metabolic dysfunction, inflammation, or experimentally induced dysbiosis. This suggests that mulberry-derived preparations may act primarily by restoring disturbed microbial ecosystems rather than by inducing large changes in already stable communities.

Taken together, the available evidence supports a model in which gut microbiota function as an intermediary between mulberry-derived compounds and host physiology. Rather than targeting a single pathway, mulberry preparations appear to influence interconnected processes involving microbial fermentation, metabolite production, intestinal barrier function, immune regulation, and host metabolic signaling. The final biological outcome depends not only on the phytochemical composition of the plant material but also on the processing history of the preparation and the physiological status of the host.

## 9. Research Gaps and Future Perspectives

Despite the growing number of experimental studies, the most important limitation of the current evidence base is the absence of well-controlled human clinical trials evaluating gut microbiota, SCFA production, intestinal permeability, and inflammatory outcomes following mulberry supplementation. Consequently, most of the mechanistic relationships discussed in this review remain supported primarily by animal models and in vitro fermentation systems. At the same time, the available evidence consistently indicates that raw material selection and processing methods substantially influence microbiota-related outcomes, suggesting that optimization and standardization of mulberry preparations should precede large-scale clinical evaluation.

Beyond the lack of clinical evidence, several additional knowledge gaps remain. Direct comparative studies evaluating *M. alba* and *M. nigra* under comparable experimental conditions are scarce, despite known differences in phytochemical composition. Many studies do not sufficiently standardize or chemically characterize extracts and fractions, making it difficult to attribute observed effects to specific groups of compounds. Furthermore, relatively few investigations directly compare extraction solvents, fermentation strategies, enzymatic modification procedures, or fractionation approaches under identical experimental conditions. Most studies also report taxonomic changes without providing strain-level resolution or functional metagenomic information, limiting mechanistic interpretation. Emerging computational approaches, including network pharmacology and molecular interaction analyses, have begun to predict microbiota-related targets linking mulberry phytochemicals with metabolic and neurodegenerative diseases. However, these hypotheses require validation using integrated experimental models combining microbiome analyses with functional and mechanistic investigations [172].

Another important consideration concerns dose dependency and safety. The available evidence suggests that biological responses do not always increase proportionally with dose. In aquaculture models, moderate inclusion of fermented mulberry leaves improved performance and gut-related parameters, whereas higher inclusion levels disrupted microbial homeostasis and impaired growth [154]. Similarly, high-dose supplementation with purified DNJ improved several barrier-related markers but was also associated with reduced villus height and decreased growth performance in rabbits [165]. These findings indicate that optimization of dosage may be as important as selection of the preparation itself.

Current toxicological evidence generally supports a favorable safety profile for mulberry-derived products. Aqueous extracts of *M. alba* leaves exhibited LD_50_ values exceeding 15 g/kg body weight and no observable adverse effects at doses up to 7.5 g/kg body weight/day, while a DNJ-standardized aqueous leaf extract showed a NOAEL of 4 g/kg body weight/day in a 28-day study [65,173]. Similarly, ethanolic extracts of *M. nigra* leaves showed no significant toxicity at doses up to 1000 mg/kg body weight/day [174]. Human studies administering DNJ-containing preparations at doses typically ranging from 18 to 36 mg DNJ/day have generally reported only mild gastrointestinal symptoms, including bloating, flatulence, and abdominal discomfort, with no serious adverse events consistently observed [175,176]. However, available safety data are derived primarily from acute and subchronic studies and provide limited information regarding long-term consumption, highly concentrated fractions, fermented preparations, and microbiota-targeted applications.

Future studies should employ chemically characterized preparations, standardized extraction and fermentation protocols, and integrated multi-omics approaches combining microbiome sequencing, metabolomics, SCFA profiling, intestinal permeability markers, and inflammatory readouts [121,123]. Particular attention should be paid to understanding how processing methods—including extraction, fermentation, enzymatic hydrolysis, purification, and formulation strategies—influence microbiota-related outcomes. Human trials should distinguish between leaf and fruit products, fermented and unfermented matrices, and standardized polysaccharide-, polyphenol-, or alkaloid-enriched preparations. Direct comparisons between *M. alba* and *M. nigra* would be especially valuable. In parallel, future work should establish optimal dosage ranges, evaluate long-term safety and clarify potential interactions with antidiabetic therapies and other medications.

Importantly, the available preclinical evidence does not suggest that all mulberry-derived preparations are biologically equivalent. Rather, the collective findings indicate that species selection, plant part, maturity stage, and processing strategy can substantially influence microbiota-related effects. Therefore, future clinical studies should be designed not only to evaluate efficacy in humans but also to identify which mulberry-derived preparations possess the greatest translational potential for supporting gut and metabolic health.

## 10. Conclusions

The available evidence indicates that mulberry-derived products can modulate gut microbiota composition and activity, influence microbial metabolite production, and affect intestinal barrier function, immune responses, and host metabolism. However, these effects are highly dependent on the botanical source, plant part, and, importantly, the processing strategy used to obtain the final preparation.

A major conclusion emerging from this review is that processing should not be viewed merely as a technical step but as a determinant of biological activity. Extraction, fermentation, enzymatic modification, purification, and formulation can substantially alter the composition and functionality of mulberry-derived products, thereby influencing their microbiota-related effects. Nevertheless, current knowledge is based predominantly on animal and in vitro studies. Well-designed human clinical trials are now needed to identify which mulberry preparations offer the greatest potential for supporting gut and metabolic health.

## Figures and Tables

**Table 1:** Main effects of mulberry leaf preparations (whole matrix, extracts, and fractions) on gut microbiota and intestinal function.

Preparation	Experimental Model	Main Microbiota and Intestinal Effects	Ref.
Whole leaf powder	Healthy mice	Modulation of fecal metabolite profiles (carbohydrate and amino acid metabolism); increased maltose, decreased glucose; minimal changes in microbial diversity and SCFA production	[130]
Methanol extract (twigs) vs. aqueous leaf extract	HFD-induced obesity in mice	Strong microbiota remodeling (methanol extract); ↓ Bacillota and *Enterococcus*; ↑ *Faecalibaculum* and *Bifidobacterium*; altered SCFA profile (↑ propionate, ↓ butyrate); highlighting effects of plant part and solvent	[125]
Aqueous leaf extract	T2D, HFD, NAFLD models	Restoration of dysbiosis; normalization of Bacillota/Bacteroidota; ↑ *Akkermansia, Bifidobacterium,* Muribaculaceae; ↓ LPS-associated taxa; ↑ SCFA; improved barrier (↑ ZO-1, occludin); ↓ inflammation (TLR9–MyD88–IFN-γ); activation of GPR43/GPR109A and AMPK; ↓ endotoxemia; modulation of endocannabinoid system	[120,121,135,136,137]
Ethanolic leaf extract	Diabetic mice	Partial normalization of dysbiosis; ↓ *Actinobacteria*; modulation of Bacteroidota and Bacillota; reversal of diabetes-associated shifts in *Bifidobacterium, Ruminococcus* 2, *Romboutsia,* and *Lactobacillus*	[136]
Leaf phenolic-rich fraction + leaf fiber fraction (1:4, *w*/*w*)	High-calorie diet induced obesity in rats	Preservation of microbial diversity; ↓ Bacillota/Bacteroidota ratio; ↓ obesity-associated Lachnospiraceae; ↑ *Lactobacillus* (incl. *L. johnsonii*); normalization of amino acid and oligopeptide metabolism; stronger anti-obesity effects than isolated fiber or polyphenol fractions, suggesting synergistic interactions between both components	[129,140]
Leaf polysaccharides	HFD-induced obesity in mice	↓ adiposity; improved insulin resistance; remodeling of microbiota; correlations with lipid metabolism; improved colonic morphology	[122]
Leaf polysaccharides	Immunosuppressed mice	Restoration of immune organs; improved barrier; ↑ SCFA (acetate, propionate, butyrate); ↑ Bacteroidota; ↓ Bacillota and selected taxa; cytokine modulation	[141]
Polysaccharide fractions (different MW/composition)	In vitro bacterial culture model	Selective stimulation of *Bacteroides* spp.; substrate-specific fermentation; ↑ acetate and propionate depending on fraction	[142]
Mulberry leaf polysaccharide fraction and its digested form	Simulated gastrointestinal digestion followed by in vitro human fecal fermentation	Digestion-dependent effects; ↓ pH; ↑ SCFA (acetate, propionate, butyrate); enhanced enzymatic inhibition; altered fermentability	[143]
Leaf oligosaccharides	Obesity/T2D models	Selective fermentation; ↑ *Lactobacillus* and *Ligilactobacillus*; ↑ lactate, acetate, butyrate; improved metabolic phenotype	[24]
Enzymatically derived leaf oligosaccharides	In vitro probiotic cultures	Resistant to simulated gastrointestinal digestion; stimulated growth of *Bifidobacterium bifidum*, *B. adolescentis*, *Lacticaseibacillus rhamnosus*, and *Lactobacillus acidophilus*; increased acetate and lactate production; demonstrated prebiotic activity	[144]
Non-digestible oligosaccharide fraction obtained by enzymatic hydrolysis of leaf polysaccharides	Fecal fermentation and animal models	Resistant to gastrointestinal digestion; increased acetate and butyrate production during fecal fermentation; enrichment of *Ligilactobacillus murinus*; associated with hypoglycaemic effects in animal models, suggesting microbiota-mediated metabolic benefits	[24,144]

DNJ—1-deoxynojirimycin; MLP—mulberry leaf polysaccharides; T2D—type-2 diabetes; HDF—high-fat diet; NAFLD—non-alcoholic fatty liver disease; SCFA—short-chain fatty acids.

**Table 2:** Main effects of mulberry fruit polysaccharides on microbiota and intestinal health.

Fraction (Model)	Key Composition/Form	Main Microbiota and Intestinal Effects	Ref.
MFS (in vitro human feces)	Arabinose, galactose, glucose, rhamnose, galacturonic acid	~45% carbohydrate consumed in 48 h; ↓ pH; large ↑ total SCFAs (acetic, propionic, butyric); shifted community toward ↑ Bacteroidetes, ↓ Firmicutes; individual sugars mapped to specific SCFAs	[128]
Purified black mulberry fruit polysaccharide (db/db mice)	Heteropolysaccharide obtained by hot-water extraction, deproteinization, decolorization, ethanol precipitation, and dialysis; MW fractions: 21–210 kDa	↓ weight gain and hyperglycemia; ↑ *Lactobacillus*, *Allobaculum*, *Bacteroides*, *Akkermansia* and Bacteroidales; improved Bacteroidota/Bacillota balance	[150]
MGO (T2DM mice)	Galacto-oligosaccharide derived from enzymatically hydrolyzed *M. nigra* fruit polysaccharides; MW: ~987 Da; galactose-rich	Improved hyperglycemia, insulin resistance and oxidative stress; ↑ Prevotellaceae and *Lactobacillus*; ↓ Lachnospiraceae; activation of hepatic PI3K/Akt signaling	[147]
MFS (HFD-induced metabolic syndrome)	*M. alba* fruit polysaccharide fraction obtained by hot-water extraction, deproteinization, and ethanol precipitation	Improved metabolic markers and colon histology; ↑ *Muribaculum* and Lachnospiraceae NK4A136 group; ↓ *Prevotella* 2, *Bacteroides*, *Faecalibacterium*, and *Fusobacterium*	[124]
Black mulberry polysaccharides (in vitro human feces)	Polysaccharides obtained by water extraction, pectinase, pectin lyase, cellulase, or mixed-enzyme extraction	All fractions fermented by microbiota and increased SCFAs; water- and pectin lyase-extracted fractions showed highest fermentability; ↑ Bacteroidota and Bacillota; ↓ Pseudomonadota and Fusobacteriota; extraction-dependent microbiota responses	[148]
MFPP (HFD-induced metabolic syndrome; conventional and FMT models)	Combined fruit polyphenol–polysaccharide fraction	Strongest improvement of MetS compared with single fractions; enrichment of *Muribaculum* and Lachnospiraceae NK4A136 group; reduction in unfavorable taxa; distinct 23-metabolite fecal signature; transferable effects demonstrated by FMT	[123,124]

MFS—mulberry fruit polysaccharides; MGO—mulberry galacto-oligosaccharide; MFPP—mulberry fruit polyphenol–polysaccharide fraction; MW—molecular weight; HFD—high-fat diet; T2DM—type 2 diabetes mellitus; FMT—fecal microbiota transplantation; SCFAs—short-chain fatty acids; MetS—metabolic syndrome.

**Table 3:** SCFA changes with different mulberry preparations.

Material and State	Main SCFA/pH Effects	Ref.
Mulberry fruit polysaccharides (fermented in vitro)	↑ acetate, propionate, butyrate; ↓ pH	[128]
Solid-fermented mulberry leaves	↑ acetic acid after 24 h	[152]
Mulberry leaf polyphenol–polysaccharide complex	Early ↑ acetate, propionate, butyrate, valerate	[167]
Fermented mulberry pomace	↑ total SCFAs and lactic acid	[22]
Fermented mulberry juice	↑ SCFAs; associated with enrichment of Bifidobacteriaceae and Lactobacillaceae	[157]
Water-, pectin lyase-, cellulase-, and enzyme-extracted fruit polysaccharides	Extraction-dependent differences in fermentability and SCFA production	[148]

SCFAs—short-chain fatty acids.

## Data Availability

No new data were created or analyzed in this study.
